# Loss of 4.1N in epithelial ovarian cancer results in EMT and matrix-detached cell death resistance

**DOI:** 10.1007/s13238-020-00723-9

**Published:** 2020-05-25

**Authors:** Dandan Wang, Letian Zhang, Ajin Hu, Yuxiang Wang, Yan Liu, Jing Yang, Ningning Du, Xiuli An, Congying Wu, Congrong Liu

**Affiliations:** 1 Department of Pathology, School of Basic Medical Sciences, Third Hospital, Peking University Health Science Center, Beijing, 100191 China; 2 Institute of Systems Biomedicine, Beijing Key Laboratory of Tumor Systems Biology, School of Basic Medical Sciences, Peking University Health Science Center, Peking University, Beijing, 100191 China; 3 College of Life Science, Zhengzhou University, Zhengzhou, 450051 China; 4 Red Cell Physiology Laboratory, New York Blood Center, New York, NY 10065 USA

**Keywords:** epithelial ovarian cancer, 4.1N, EMT, anoikis, entosis

## Abstract

Epithelial ovarian cancer (EOC) is one of the leading causes of death from gynecologic cancers and peritoneal dissemination is the major cause of death in patients with EOC. Although the loss of 4.1N is associated with increased risk of malignancy, its association with EOC remains unclear. To explore the underlying mechanism of the loss of 4.1N in constitutive activation of epithelial-mesenchymal transition (EMT) and matrix-detached cell death resistance, we investigated samples from 268 formalin-fixed EOC tissues and performed various *in vitro* and *in vivo* assays. We report that the loss of 4.1N correlated with progress in clinical stage, as well as poor survival in EOC patients. The loss of 4.1N induces EMT in adherent EOC cells and its expression inhibits anoikis resistance and EMT by directly binding and accelerating the degradation of 14-3-3 in suspension EOC cells. Furthermore, the loss of 4.1N could increase the rate of entosis, which aggravates cell death resistance in suspension EOC cells. Moreover, xenograft tumors in nude mice also show that the loss of 4.1N can aggravate peritoneal dissemination of EOC cells. Single-agent and combination therapy with a ROCK inhibitor and a 14-3-3 antagonist can reduce tumor spread to varying degrees. Our results not only define the vital role of 4.1N loss in inducing EMT, anoikis resistance, and entosis-induced cell death resistance in EOC, but also suggest that individual or combined application of 4.1N, 14-3-3 antagonists, and entosis inhibitors may be a promising therapeutic approach for the treatment of EOC.

**Electronic supplementary material:**

The online version of this article (10.1007/s13238-020-00723-9) contains supplementary material, which is available to authorized users.

## Introduction

Ovarian cancer is the leading cause of death from gynecologic cancers globally, with 13,980 deaths occurring accounting for about 5% of total cancer deaths in females (Siegel et al., 2019). Epithelial ovarian cancer (EOC) comprises 90% of all ovarian cancers and most patients diagnosed with advanced EOC have an overall 5-year survival rate of less than 30% (Bast et al., 2009; Armstrong et al., 2019). Unlike other tumors, peritoneal dissemination is the main metastatic process of EOC, rather than the direct extension of the tumor into adjacent tissues and lymphatic dissemination. Peritoneal dissemination generally leads to a sharp rise in the clinical stage (Kipps et al., 2013). Thus, there is an urgent need to discover the mechanism of peritoneal dissemination, as well as to find useful biomarkers and effective therapeutic targets for EOC treatment.

Protein 4.1N is encoded by the *EPB41L1* (erythrocyte membrane protein band 4.1 like 1) gene (Baines et al., 2014), and is widely expressed in many cells and tissues, being especially abundant in brain, spinal cord, and adrenal glands (Walensky et al., 1999; Taylor-Harris et al., 2005). As a major human cell membrane cytoskeleton cross linker protein, 4.1N links the plasma membrane to cytoskeletal structures at specific cellular locations by directly binding partner proteins and/or phosphoinositides through its FERM domain (Chishti et al., 1998). In addition, 4.1N acts as a protein partner to regulate the activation of focal adhesion kinase (FAK), which binds to cellular partners and functions in the cell cortex at sites of integrin-mediated cell attachment to the extracellular matrix (ECM) (Frame et al., 2010). Furthermore, 4.1N plays a vital role in nuclear export signals (Frame et al., 2010) and nuclear assembly (Krauss et al., 2003). A series of investigations indicated that 4.1N acts as a tumor suppressor by disrupting cancer cell proliferation and migration, as well as inhibiting tumor occurrence and development in non-small cell lung cancer (Yang et al., 2016; Wang et al., 2016), breast cancers (Ji et al., 2012), and EOC (Zhang et al., 2016). Our previous investigation confirmed that the decrease or loss of 4.1N expression was highly common in ovarian cancer cell lines and tissues; EOC patients with 4.1N down-regulation showed an increased risk of tumor malignancy, including ascites, intraperitoneal dissemination, poor histological differentiation, and short progression-free survival (PFS) (Xi et al., 2013). By changing the cellular localization of hypoxia‑induced factor 1α (HIF-1α), 4.1N can inhibit hypoxia-induced epithelial-mesenchymal transition (EMT) in EOCs (Zhang et al., 2016). These results implied that 4.1N plays a crucial role in inhibiting tumor occurrence and development in EOC; however, little is known about the precise molecular mechanisms underlying the role of 4.1N on EOC progression.

As a typical course of metastasis of tumors, unlike hematogenous metastasis and lymphatic metastasis, which serve as two-dimensional (2D) metastasis mechanisms that provide a tube-like track for the rapid intravascular dissemination of cancer cells, peritoneal dissemination is a multistep biological process involving transition from three-dimensional (3D) metastasis to 2D metastasis (Friedl and Alexander, 2011). Initially, epithelial basement membranes display a 3D track system, cancer cells need to undergo the EMT and acquire a migratory and invasive phenotype that allows it to leave the primary tumor. Next, cancer cells detach from the basement membrane and enter the abdominal cavity, a typically 2D cell surface. Adherent cells face the problem of “anoikis”; however, if cells survive, adhere, and proliferate in the new environment, a secondary tumor site can be formed (Simpson et al., 2008). Anoikis is a unique form of detachment-induced cell death induced by the detachment of epithelial cells from the ECM (Meredith et al., 1993; Frisch and Francis, 1994; Frisch and Screaton, 2001). It is considered a key mechanism for preventing the colonization of distant organs by preventing adherent-independent cell growth and attachment to an inappropriate matrix. Resistance to anoikis indicates a failure to execute the anoikis program, leading to the survival of anchorage-independent cells in suspension (Taddei et al., 2012; Paoli et al., 2013). Notably, another form of cell death exists in matrix-detached cells, known as “entosis”, which is described as a non-apoptotic cell death program induced by a complete cell invasion into and residing in the cytoplasm of a neighboring host cell, forming a cell-in-cell structure. Entosis is most commonly observed in a variety of human malignancies, but there are few reports describing the molecular mechanisms underlying entosis in malignancy disease (Overholtzer et al., 2007). In addition, the mechanism of entosis involvement in peritoneal dissemination in EOC is unclear. In terms of the importance of peritoneal dissemination on the migratory and invasive potential of EOC, a precise understanding of the processes involved in EMT and matrix-detached cell death resistance is vital.

In this study, our investigation revealed that the loss of 4.1N was correlated with increased clinical stage progression, as well as poor overall survival (OS) and PFS in EOC patients. Through *in vitro* studies, we demonstrated that the expression of 4.1N not only down-regulated the expression of EMT-related markers and inhibited migration and invasiveness in adherent EOC cells, but also inhibited anoikis resistance and EMT by directly binding and accelerating the degradation of 14-3-3 in a cell suspension. Furthermore, 4.1N expression decreased the rate of entosis, thereby inhibiting cell death resistance in the EOC cell suspension. In contrast, the loss of 4.1N aggravated EMT and matrix-detached cell death resistance. Xenograft tumors in nude mice also showed that the loss of 4.1N aggravated the peritoneal dissemination of EOC cells. Single-agent and combination therapies with a ROCK inhibitor and a 14-3-3 antagonist were shown to reduce tumor spread to different degrees. Thus, individual or combined application of 4.1N, 14-3-3 antagonists, and entosis inhibitors may be a promising therapeutic approach for the treatment of EOC.

## Results

### Loss of 4.1N exacerbates EOC aggressiveness

**Figure 1:**
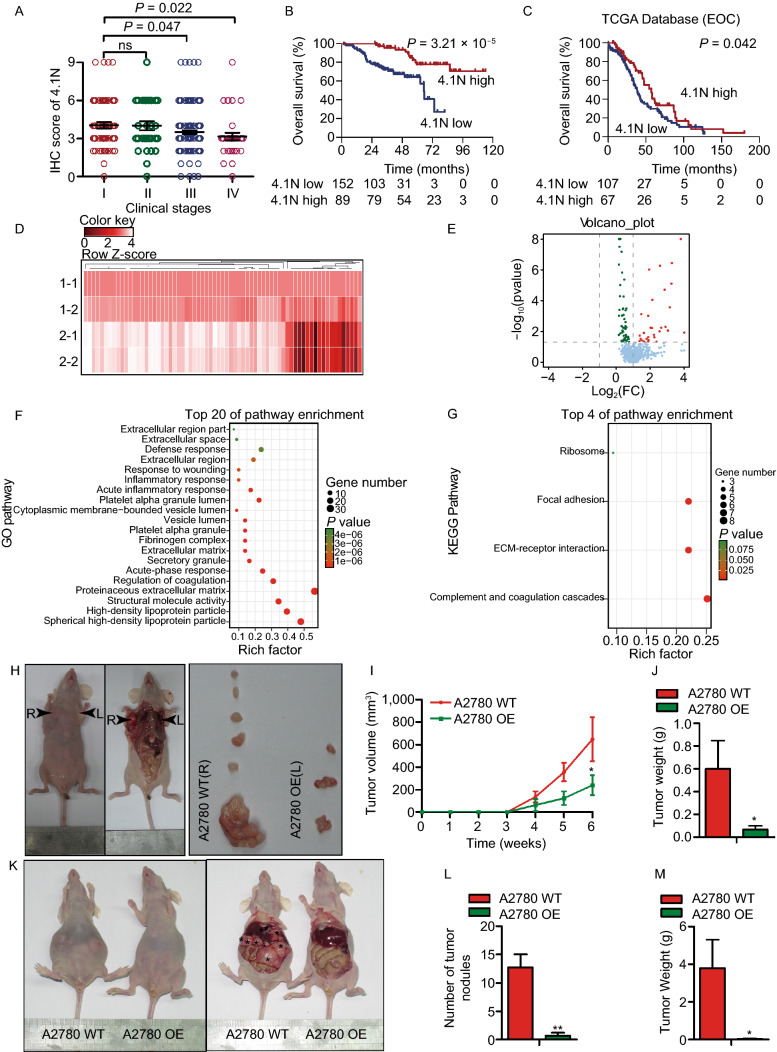
**Loss of 4.1N induces aggressiveness** ***in vivo*** **and EOC patients**. (A) IHC staining score of 4.1N expression in human EOC (clinical stages I–IV). (B and C) Kaplan-Meier OS curves in our data and TCGA database for patients with EOC stratified by low and high protein expressions of 4.1N (*P* = 3.21 × 10^−5^ and 0.042, respectively). The heatmap (D) and volcano plot (E) of the 89 proteins (more than 0.5-fold change and *P* < 0.05) (1-1/1-2: 4.1N normal expression tissue samples; 2-1/2-2: 4.1N loss expression tissue samples), proteins highlighted in red are significantly increased with 4.1N, proteins highlighted in green are decreased with 4.1N. (F) GO pathway analysis and (G) KEGG pathway analysis were used to look at the canonical pathways enriched due to differentially 4.1N expression and rich factors and gene numbers are represented in the figures. (H) Xenograft model in BALB/c nude mice, representative images of tumors from all mice in each group. Tumor volume (I) and tumor weights growth curves (J) for tumors formed by the indicated cells. (K) Macroscopical morphology of metastatic tumor nodules were found in abdominal cavity of mice as shown in the black arrows, including in intestine, mesentery, liver, and spleen. (L and M) The number and weight of dissected metastatic tumor nodules were quantified. All data are presented as mean ± SD. *P < 0.05, **P < 0.01 (Student’s *t*-test)

We examined 268 archived paraffin-embedded EOC samples and found a negative correlation between 4.1N expression and the clinical stage of EOC patients (Figs. S1A and 1A). The median OS for patients with low 4.1N expression (31.47 months) was significantly lower than that for patients with high 4.1N expression (55.07 months; *P* = 3.21 × 10^−5^) (Fig. 1B); a similar tendency (54.63 months vs. 55.07 months) was also observed in terms of PFS (*P* = 3.53 ×10^−10^) (Fig. S1D). The Cancer Genome Atlas (TCGA) database analysis revealed that patients with high 4.1N expression consistently showed an increased OS and PFS compared to those in patients with low 4.1N expression (*P* = 0.042 and *P* = 0.048) (Figs. 1C and S1C).

Subsequently, four clinical EOC samples were randomly selected to detect protein expression—two samples with low 4.1N expression, and two with high 4.1N expression. A total of 89 proteins displayed significantly different expression levels between the two groups (*P* < 0.05, absolute fold change ≥ 0.5) (Fig. 1D and 1E). A series of proteins showed a significant increase in the expression of 4.1N including RAB5B, vitronectin, thrombospondin, and fibronectin. In addition, many proteins showed a significant decrease in 4.1N-expression tissue, including desmin, metadherin, and collagen-related proteins (Table S4). Complement and coagulation cascades, ECM-receptor interaction, and focal adhesion were then identified as the top three terms identified by Kyoto Encyclopedia of Genes and Genomes (KEGG) analysis; some extracellular-related pathways, including extracellular region, extracellular region part, extracellular matrix, proteinaceous extracellular matrix, and extracellular space (Fig. 1F and 1G), were likewise revealed by Gene Ontology (GO) pathway analysis.

To study the effect of 4.1N expression on the EOC cell lines *in vivo*, A2780 overexpressing (OE) cells stably transfected with the 4.1N gene (2 × 10^6^) and A2780 WT cells with endogenous loss 4.1N expression (2 × 10^6^) were injected into the left flank and right flank of immune-deficient nude mice, respectively. Six weeks after injection, we observed that the tumors formed by 4.1N-expressing cells were significantly smaller than those formed by the control cells (Fig. 1H–J). Meanwhile, A2780 OE cells (5 × 10^6^) or A2780 WT cells (5 × 10^6^) were injected into the abdominal cavity of nude mice. Compared to the control group, the number of tumor nodules (*P* = 0.002) and the tumor weight (*P* = 0.034) of the intra-abdominal metastatic foci were significantly lower in A2780 OE-injected group (Fig. 1K–M). Thus, the loss of 4.1N expression is related to a poor prognosis in EOC patients and increased EOC metastasis *in vivo*.

### Loss of 4.1N promotes EOC cells EMT *in vitro*

To investigate the effect of decreasing 4.1N levels on EOC progression, we selected two EOC cell lines: SKOV3, which endogenously expresses 4.1N; and A2780, which lacks 4.1N expression (Xi et al., 2013). We generated 4.1N-overexpressing stable cell lines in A2780 cells (referred to as A2780 OE) to compare with the wild type 4.1N-silenced A2780 cells (A2780 WT). Meanwhile, we knocked out endogenous 4.1N in SKOV3 cells (SKOV3 KO) to compare with the 4.1N-expressing SKOV3 cells (SKOV3 WT) (Fig. S1E and S1F).

**Figure 2:**
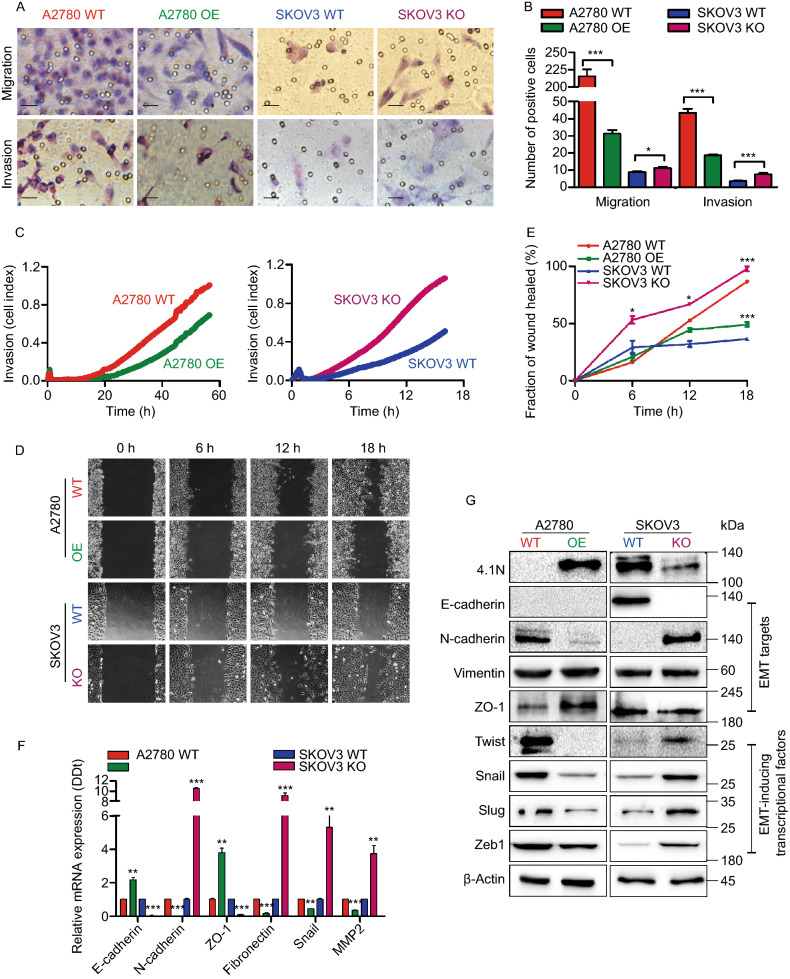
**Loss of 4.1N promotes EOC cells EMT** ***in vitro***. (A) Representative images of transwell migration assay (top) and transwell invasion assay (bottom) of A2780 cells and SKOV3 cells with 4.1N-expression and 4.1N-loss. (B) Graphical representation of transwell migration assay and transwell invasion assay. The transwell migration assay of A2780 OE cells (31.25 ± 6.11) showing reduction in transwell migration, compared to empty vector control (215.63 ± 27.44) (*n* = 8, 2-tailed unpaired *t*-test, *P* < 0.001). The transwell migration assay of SKOV3 KO cells (11.13 ± 2.17) showing increase in transwell migration, compared to empty vector control (8.75 ± 1.83) (*n* = 8, 2-tailed unpaired *t*-test, *P* = 0.033). The transwell invasion assay of A2780 OE cells (18.63 ± 1.77) showing reduction in transwell invasion, compared to empty vector control (43.63 ± 6.07) (*n* = 8, 2-tailed unpaired *t*-test, *P* < 0.001). The transwell invasion assay of SKOV3 KO cells (7.50 ± 1.85) showing increase in transwell invasion, compared to empty vector control (3.50 ± 0.76) (*n* = 8, 2-tailed unpaired *t*-test, *P* < 0.001). All these means ± SD results from three independent experiments. Scale bar, 50 µm. (C) Representative graph of the invasion curve using real-time cell analyzer (RTCA). The rate of invasion was monitored in real-time using the xCELLigence system (*n* = 4). (D) Representative images of scratch wound healing assay of A2780 cells and SKOV3 cells with expression of 4.1N and respective control. (E) Graph of scratch wound healing assay of A2780 cells and SKOV3 cells with 4.1N-expression and 4.1N-loss. A2780 OE cells showing 37.7% reduced wound area, compared to control wound area (*n* = 3, 2-tailed unpaired *t*-test, *P* < 0.05); compared to SKOV3 KO cells, SKOV3 KO cells indicate 61.5% increase (*n* = 3, 2-tailed unpaired *t*-test, *P* < 0.05). (F) Effects of 4.1N on EMT-related mRNA expression was assayed by qRT-PCR and results means ± SD from three independent experiments. β-Actin was used as internal control. (G) Effects of 4.1N on EMT-related proteins expression was assayed by Western blot. β-Actin was loading control. *P < 0.05, **P < 0.01, ***P < 0.001

EOC cell invasion initially involves trans-basement migration, followed by survival in the abdominal cavity, and ending with dissemination at the metastatic sites. We investigated the effect of 4.1N loss on the EMT, which is a critical step for EOC cells to transition from a 3D environment into 2D metastasis. qRT-PCR analysis showed an approximately 1.2-fold increase in E-cadherin expression, 2.8-fold increase in ZO-1 expression, 32.3-fold decrease in N-cadherin, 4.9-fold decrease in Fibronectin, 1.3-fold decrease in Snail, and 1.9-fold decrease in MMP2 in A2780 OE cells; while 4.1N depletion in SKOV3 cells revealed an approximately 9.6-fold increase in N-cadherin, 8.1-fold increase in Fibronectin, 4.3-fold increase in Snail, 2.7-fold increase in MMP2, 32.3-fold decrease in E-cadherin, and 10.1-fold decrease in ZO-1 expression (Fig. 2F). Western blot confirmed that the loss of 4.1N led to increased N-cadherin, Twist, Snail, Slug, and Zeb-1 expression (Fig. 2G). Next, we performed a transwell migration assay and Matrigel invasion assay to monitor cell motility and invasiveness. A2780 OE cells showed a 5.7-fold decrease in migration and a 1.3-fold decrease in invasion compared with A2780 WT cells. Meanwhile, SKOV3 cells revealed a 0.3-fold increase in migration and a 1.1-fold increase in invasion when 4.1N was depleted (Fig. 2A and 2B). A would healing assay and real-time invasion assay using RTCA further confirmed that the loss of 4.1N enhanced EOC cell migration and invasion (Fig. 2C–E). These results indicate that the loss of 4.1N enhances EOC cell migration and invasion and positively modulates the expression of the above EMT-related markers.

### 4.1N maintains anoikis sensitivity in EOC cells

**Figure 3:**
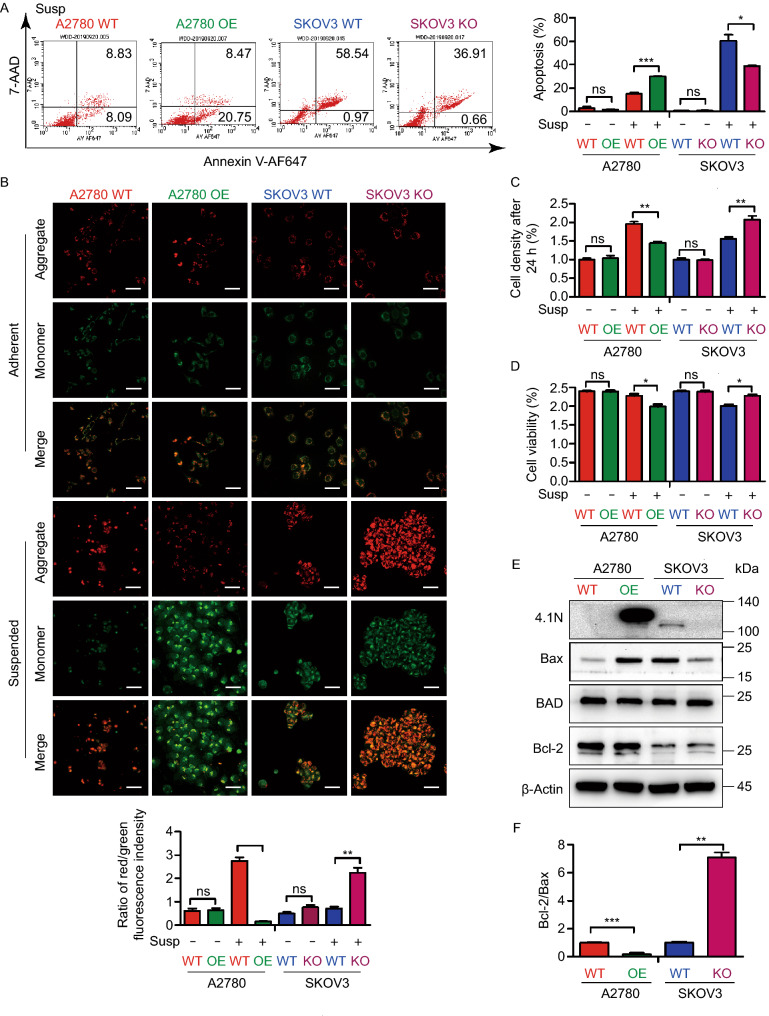
**Loss of 4.1N promotes anoikis resistance of EOC cells** ***in vitro***. (A) Representative images of flow cytometry results with 7AAD–Annexin V-AF647 staining, A2780 WT, A2780 OE, SKOV3 WT and SKOV3 KO cells in suspension (left); results means ± SD from three independent experiments (right). (B) Representative images of A2780 WT, A2780 OE, SKOV3 WT and SKOV3 KO cells subjected to JC-1 staining assays (top); The ratio of red/green fluorescence indensity of JC-1 assay was representation on graphical (bottom). Scale bar, 50 µm. (C) Proliferation rate of anoikis resistant A2780 WT, A2780 OE, SKOV3 WT and SKOV3 KO cells was analyzed by comparing cell densities after 24 h incubation. (D) Cell viability was first evaluated using the trypan blue assay. (E) Effects of 4.1N on apoptosis-related mRNA assayed by qRT-PCR and results means ± SD from three independent experiments. β-Actin was used as internal control. (F) Effects of 4.1N on apoptosis-related proteins expression was assayed by Western blot. β-Actin was loading control. *P < 0.05, **P < 0.01, ***P < 0.001

Subsequently, we analyzed the survival of EOC cells in the abdominal cavity. We first analyzed the role of 4.1N on EOC cell apoptosis by flow cytometry. In adherent cells, there was no significant change in apoptosis rate (Fig. 3A, S2F, and S2G). When EOC cells enter a 2D environment in the abdominal cavity, anoikis becomes a major challenge to their survival. We next assayed cell survival in suspension as a readout of their ability to withstand cell death upon loss of adhesion. Interestingly, in suspension culture, A2780 OE cells demonstrated a 1.0-fold increase and SKOV3 KO cells showed a 0.6-fold decrease in apoptosis, compared with control cells (Fig. 3A, S2G, and S2H). A similar tendency was also observed in cell viability (Fig. 3D and S2I), indicating that the loss of 4.1N promotes anoikis resistance in EOC cells. Our mitochondrial function analysis, demonstrated by JC-1 staining, further confirmed these findings; in A2780 and SKOV3 cells expressing 4.1N showed reduced red fluorescence and elevated green fluorescence, indicating that 4.1N expression induced mitochondrial dysfunction in EOC cells (Fig. 3B).

Furthermore, we compared the relative proliferation rates of anoikis-resistant cell pairs (A2780 OE vs. A2780 WT, and SKOV3 KO vs. SKOV3 WT) by re-plating and culturing an equal number of each cell type in a cell culture dish for 24 h, followed by cell counting. Our results revealed that anoikis-resistant cells without 4.1N expression exhibited a 0.3-fold increase of proliferation rates relative to those with low 4.1N expression (Fig. 3C). Consistent with the observed association between 4.1N expression and susceptibility to anoikis, 4.1N-expressing cells expressed higher levels of Bax, but lower levels of Bcl-2, in suspension (Fig. 3E and 3F). These data suggest that 4.1N promotes anoikis sensitivity in EOC cells.

### Loss of 4.1N promotes entosis in EOC cells

**Figure 4:**
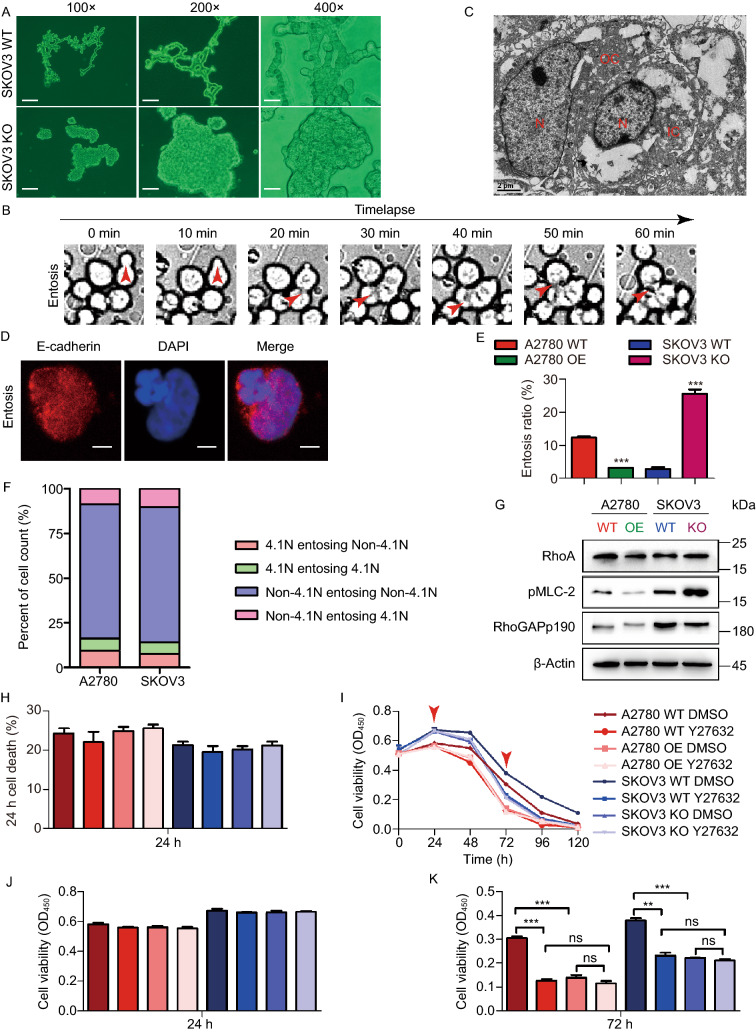
**Loss of 4.1N promotes entosis in EOC cells**. (A) Representative images of SKOV3 WT and SKOV3 KO using an inverted microscope at 100×, 200× and 400× magnification after 24 h incubation with suspension. Scale bar, 200 µm, 100 µm and 50 µm, respectively. (B) SKOV3 KO cells cultured in suspension were analyzed by timelapse microscopy, images were collected every 10 min (See Movie S1). Scanning electron microscope photomicrograph (×6,000) (C) and a laser scanning confocal microscope (D) show representative images of entosis structures. Scale bar, 2 µm and 10 µm, respectively. (E) The formation ratio of entosis in different cell lines. Cells were cultured in the absence of matrix adhesion for 6 h before analysis. Data are mean ± SD of three or more fields with >400 cells analyzed for each cell line. (F) Graph shows calculation for cell entosis events to four subcategories as indicated. (G) RhoA, pMLC-2, p190RhoGAP proteins assayed by Western blot. β-Actin was loading control. (H) Cell death rate were counted by PI staining. (I) A2780 WT, A2780 OE, SKOV3 WT and SKOV3 KO cells (10,000 cells/96 well) in suspension treated Y27632 at 40 μmol/L, cell viability measured by CCK8 at 0, 24, 48, 72, 96, 120 h; presented as means ± SD from three separate experiments. Graphical representation of cell viability at 24 h (J) and 72 h (K). *P < 0.05, **P < 0.01 and ***P < 0.001 (Student’s *t*-test)

To explore the role of 4.1N in cell death in greater detail, we monitored live cell behavior in suspended EOC cells in real time by time-lapse microscopy. Interestingly, we discovered that 4.1N-expressing cells displayed cord-like connections, while 4.1N-non-expressing cells were clumped together in suspension (Fig. 4A). Strikingly, we observed that at 2–4 h after cell detachment, 4.1N-non-expressing cells started to internalize their neighboring cells (Fig. 4B and Movie S1). Full internalization was achieved within several hours. We observed cases where one 4.1N-non-expressing cell continuously internalized neighboring cells in sequence (Fig. 4B and Movie S1). This cell internalization phenomenon has been reported and termed entosis, which frequently occurs in matrix-detached cells and has been described as a nonapoptotic cell death program (Overholtzer et al., 2007). Confocal microscopy and scanning electron microscopy images of suspended SKOV3 KO cells showed that the internalized cells had a round shape with a large vacuole, while the internalizing cell exhibited a crescent shape, consistent with previously reported entositic cellular structures (Mackay and Muller, 2019) (Fig. 4C and 4D).

Interestingly, A2780 OE cells demonstrated a 2.8-fold decrease in cell entosis rate compared with that of the control, and 4.1N knockdown in SKOV3 cells resulted in a 7.9-fold increase (Fig. 4E). Previous studies have indicated that entosis is dependent on Rho-ROCK activity (Overholtzer et al., 2007). Accordingly, we found that loss of 4.1N expression increased the levels of RhoA and phosphorylated myosin light chain 2 (Fig. 4G). Moreover, treatment with the ROCK inhibitor Y27632 exerted dose-dependent inhibition effects on entosis in 4.1N-non-expressing cells (Fig. S2A).

Next, we examined the role of entosis in cell proliferation and cell death. While the short-term cell proliferation and cell death rates remained unchanged after entosis inhibition (Fig. 4H and S2B), we observed the death of a large number of cells after 48 h in 4.1N-expressing cells, but not in 4.1N-non-expressing cells, and treatment with an entosis inhibitor reversed this phenomenon (Fig. 4I–K). These results suggested that the loss of 4.1N may increase cell death resistance by promoting entosis. Increasing evidence shows that malignant cells can outcompete benign cells by entosing and retrieving nutrients from them (Kroemer and Perfettini, 2014). To detect whether 4.1N-non-expressing cells could outcompete 4.1N-expressing cells via entosis, we labeled A2780 OE and SKOV3 WT cells with CellTracker Green CMFDA, labeled A2780 WT and SKOV3 KO cells with CellTracker Red CM-Dil, and performed co-culture experiments in suspension. Interestingly, only 4.1N-non-expressing cells could actively engulf 4.1N-non-expressing cells (Fig. 4F). These results revealed that although 4.1N-non-expressing cells cannot outcompete 4.1N-expressing cells, loss of 4.1N cells though internal group entosis could increase cell death resistance.

### 4.1N negatively regulates 14-3-3 expression

**Figure 5:**
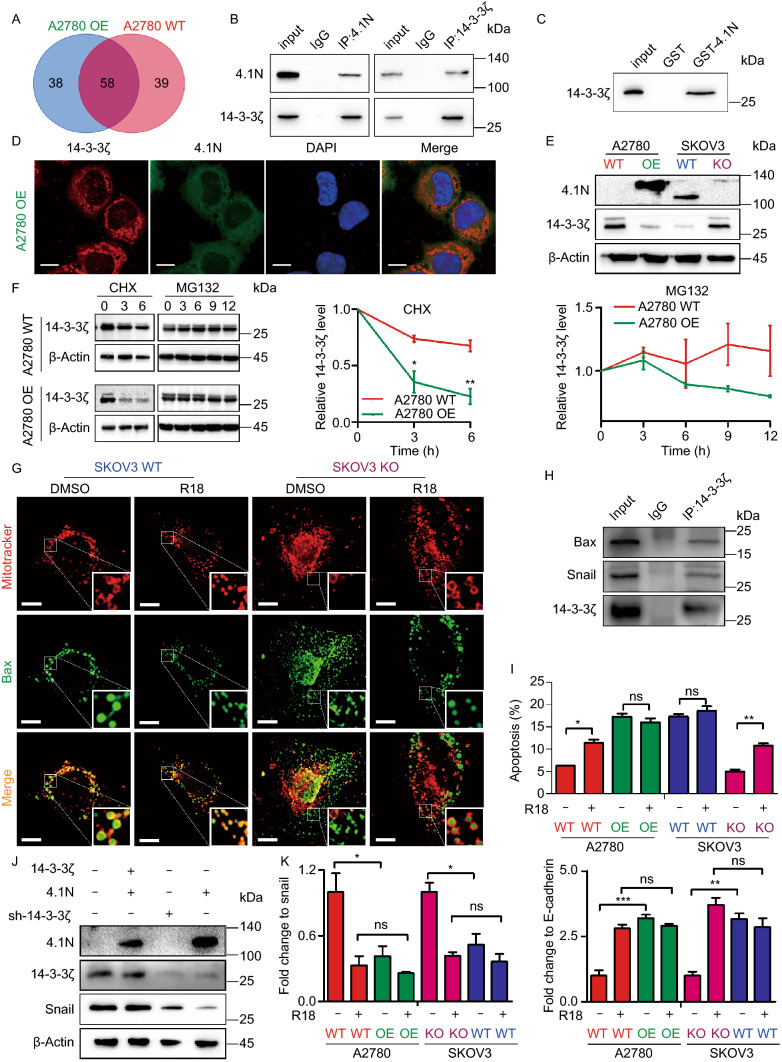
**4.1N negatively regulate 14-3-3 expression**. (A) Veen diagram showing the interaction proteins of 4.1N in A2780 WT and A2780 OE cells using Co-IP/MS, then, we use Co-IP assay (B) and GST-Pulldown assay (C) to validated. (D) Subcellular localization of 4.1N, 14-3-3ζ and DAPI in A2780 OE cells. Scale bar, 10 µm. (E) Western blot assay detected the protein expression effect of 4.1N on 14-3-3ζ. β-Actin was used to serve as a loading control. (F) 14-3-3ζ protein expression in A2780 WT and A2780 OE cells was determined with western blot analysis following treatment with the translation inhibitor CHX for 0, 3, 6 h and the proteasome inhibitor MG‐132 for 0, 3, 6, 9, 12 h. β-Actin was used to serve as a loading control. (G) The co-staining of mitochondria and Bax. In the enlarge panel, the yellow fluorescence is the foci of mitochondria and Bax, indicative of apoptosis activation. The loss of 4.1N could limit the overlap of mitochondria and Bax, however, R18 can reverse it. Scale bar, 10 µm. (H) Co-IP assay of 14-3-3ζ by agarose beads followed by Western blot with indicated antibodies. (I) Flow cytometer assay was used to quantify R18 effect on cellular apoptosis. (J) Effects of 4.1N and 14-3-3ζ on snail proteins was assayed by Western blot. β-Actin was used to serve as a loading control. (K) qRT-PCR assay was used to quantify R18 effect on EMT-related mRNA expression and results means ± SD from three independent experiments. β-Actin was used as internal control

To gain further insights into the functions of 4.1N, we performed Co-IP/LC-MS/MS analysis to identify 4.1N-interacting proteins. 14-3-3ζ/δ, 14-3-3γ, and 14-3-3η were found to interact with 4.1N, and 4.1N protein–protein interaction networks based on the Biogrid database also indicated interactions between 4.1N and the 14-3-3 protein family (Figs. 5A, S3A and Table S3). Further Co-IP, GST pull-down, and immunofluorescence assays also verified the association between 4.1N and 14-3-3ζ (Fig. 5B–D). Gene correlation analysis using the R2 platform revealed that 4.1N expression was negatively correlated with 14-3-3ζ expression (R = −0.157, *P* = 3.0 × 10^−4^; Fig. S3B). We also identified a negative correlation between 4.1N and 14-3-3ζ expression at both the mRNA and protein levels (Figs. 5E, S2C, and S2D). Moreover, we found that 4.1N accelerated 14-3-3ζ degradation (Fig. 5F). 14-3-3 has been reported to play a crucial role in regulating apoptosis and EMT (Hou et al., 2010; Nomura et al., 2015); thus, we hypothesized that the increase in tumor aggressiveness upon the loss of 4.1N may be mediated through 14-3-3ζ. Initially, we identified that 14-3-3ζ interacted with Bax (Fig. 5H). Furthermore, 14-3-3 blocked Bax localization to the mitochondria by restricting Bax to the cytoplasm. Treatment with a 14-3-3 antagonist was found to reverse this change in the subcellular localization of Bax (Fig. 5G). Similarly, treatment with a 14-3-3 antagonist showed an approximately 1-fold increase in 4.1N-non-expressing EOC cells (Fig. 5I). In addition, by interacting with and up-regulating the expression of the key EMT-related transcription factor Snail, 14-3-3ζ promoted EOC cell EMT; this was reversed by 14-3-3 antagonist treatment (Fig. 5J and 5K). These findings suggest that 4.1N reduces EOC aggressiveness through negatively regulating 14-3-3 *in vitro*.

### Loss of 4.1N induces EOC aggressiveness via 14-3-3 expression and entosis *in vivo*

**Figure 6:**
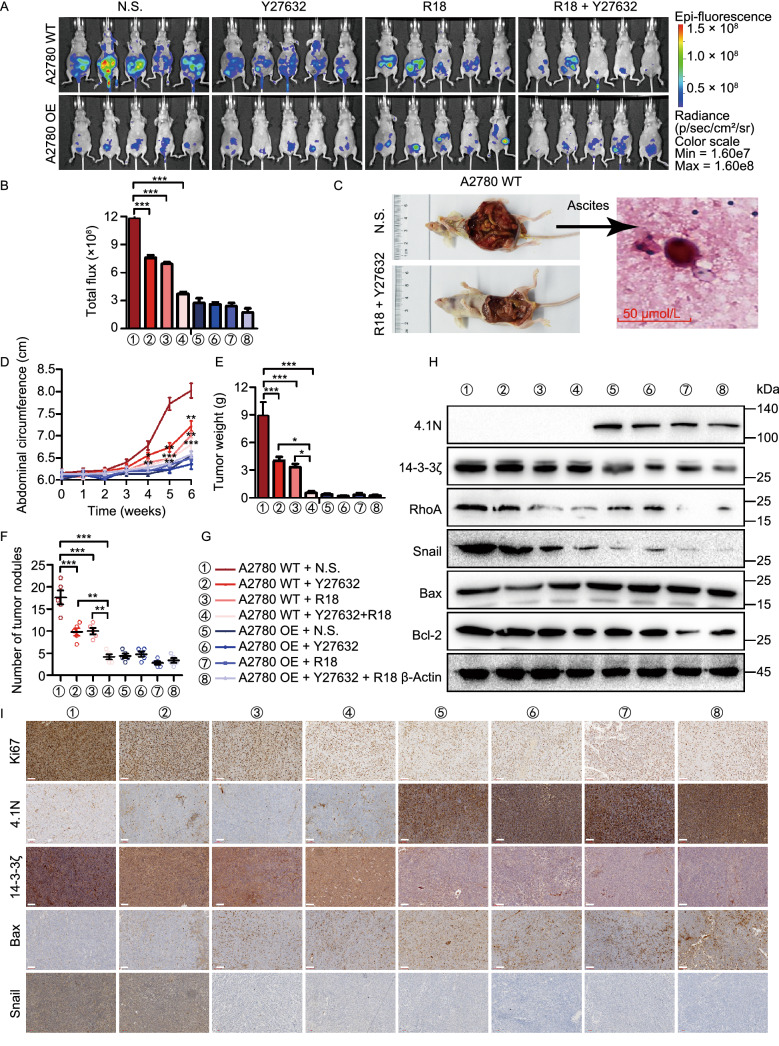
**Loss of 4.1N induces EOC aggressiveness via 14-3-3 expression and entosis *in vivo***. Peritoneal dissemination nude mouse models were established using A2780 WT and A2780 OE cells treatment with Y27632 and (or) R18. (A and B) *In vivo* bioluminescent images and quantification of 6 weeks after abdominal injection indicated cells (*n* = 5, two-sided Student’s *t*-test). (C) H&E stained sections of abdominal ascites containing examples of entosis structures, as indicated by arrows. Scale bars = 50 μm. Abdominal circumference curves (D), the weight (E) and number (F) of dissected metastatic tumor nodules were quantified. (G) Different colors and number indicate different groups. (H) Protein expression of 4.1N, 14-3-3ζ, RhoA, Snail, Bax and Bcl-2 in nude mouse tissue detected by Western blot. β-Actin was used as an endogenous control. (I) Protein expression of Ki67, 4.1N, 14-3-3ζ, Bax and Snail in nude mouse tissue detected by IHC. Scale bar, 100 µm. *P < 0.05, **P < 0.01 and ***P < 0.001 (Student’s *t*-test)

To confirm the above *in vitro* findings showing that the loss of 4.1N induces EMT and anoikis resistance via 14-3-3, as well as the activation of Rho-ROCK activity and the promotion of entosis, the effects of single-agent and combined therapies using the ROCK inhibitor Y27632 and the 14-3-3 antagonist R18 were evaluated in peritoneal dissemination nude mouse models. Drug treatment was well tolerated in the peritoneal dissemination models, with no apparent toxicity. Mice bearing A2780 WT cell xenografts treated with Y27632 or R18 alone showed a significant reduction in the abdominal circumference of the tumor, and a reduction in the final tumor nodules and weight determined by necropsy (Fig. 6D–G, S1G, and S1I). This reduction in tumor nodules was significantly greater in mice treated with the combination therapy than after treatment with either drug alone (Fig. 6D–G, S1G, and S1I). Bioluminescent imaging and the obtained average radiance values indicated a statistically significant inhibition of tumor nodule dissemination in single-agent and combination therapies with Y27632 and R18; necropsy assessments confirmed this observation (Fig. 6A and 6B). After the intraperitoneal inoculation of A2780 OE and A2780 WT cells, no mice (0/32) in the A2780-OE group formed ascites, while all eight mice (8/8) in the A2780-WT group showed varying degrees of ascites formation (Fig. S1H). Single-agent or combination therapy with Y27632 and R18 in A2780 WT mice reduced the formation of ascites to a limited extent; however, entotic engulfment was identified in ascites smear (Fig. 6C). In A2780 WT mice treated with Y27632 and R18, the protein levels of RhoA, Bcl-2, and Snail were decreased, while that of Bax was increased (Fig. 6H and 6I). These results indicate that 4.1N reduces EOC aggressiveness by negatively regulating 14-3-3 expression and inhibiting entosis *in vivo*.

### Clinical relevance of 4.1N loss-induced entosis, 14-3-3-dependent EMT and anoikis resistance in EOC

**Figure 7:**
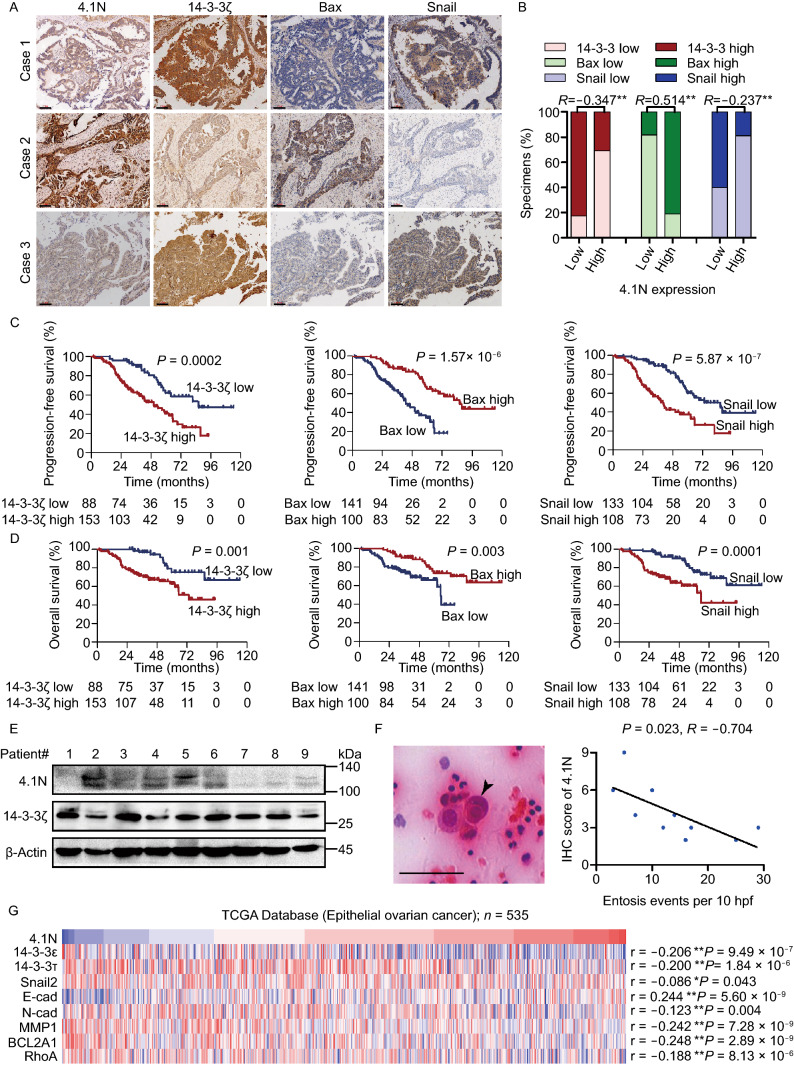
**Clinical relevance of 4.1N loss-induced entosis, 14-3-3-dependent EMT and anoikis resistance in EOC**. (A and B) 4.1N were associated with 14-3-3ζ, Bax and Snail molecules expression in 268 primary human EOC specimens. Scale bar, 100 μm. (C) Kaplan-Meier PFS curves of patients with EOC with low versus high expression of 14-3-3ζ, Bax and Snail; (*P* = 0.0002, *P* = 1.57 × 10^−6^ and *P* = 5.87 × 10^−7^, respectively); (D) Kaplan-Meier OS curves of patients with EOC with low versus high expression of 14-3-3ζ, Bax and Snail; (*P* = 0.001, *P* = 0.003 and *P* = 0.0001, respectively). (E) 4.1N and 14-3-3ζ expression at the protein level was detected by western blot analysis in nine EOC tissues. β-Actin was used as an endogenous control. (F) H&E stained sections of abdominal ascites containing examples of entosis structures, as indicated by arrows. Scale bars = 50 μm (left); Correlation between abdominal ascites slides entosis occurrence and corresponding tissues 4.1N IHC scores (*n* = 10, Spearman correlation; *P* = 0.023, r = −0.704) (right). (G) 4.1N mRNA expression was correlated positively with E-cadherin mRNA expression and negatively with 14-3-3ε, 14-3-3τ, Snail2, N-cadherin, MMP1, BCL2A1 and RhoA mRNA expression in published profiles of ESCC (*n* = 535, *P* < 0.05; TCGA database of ovarian carcinoma)

Finally, we examined whether 4.1N, 14-3-3ζ, Bax, and Snail were clinically relevant. EOC tissue specimens showed that 4.1N expression was positively correlated with Bax expression and negatively correlated with 14-3-3ζ and Snail expression (Figs. 7A, 7B, and S1B). Patients with reduced Bax and increased 14-3-3ζ and Snail expression generally showed a shorter OS (Fig. 7C); a similar tendency was also demonstrated in PFS curves (Fig. 7D). As shown in Fig. 7E, in nine cases, 4.1N expression was negatively correlated with 14-3-3ζ expression in EOC patient tissues. Representative images of entosis in EOC patients’ ascites sediment showed that the internalizing cell had a crescent shape and the internalized cell had a rounded shape (Fig. 7F). We next investigated the rate of entosis by measuring the number of entosis events per 10 high-power fields (HPF) from a smear of ascites, and the immunohistochemistry (IHC) score of 4.1N in corresponding patient tissues. Our data indicated a significant negative link between 4.1N protein expression and entotic engulfment events (Fig. 7F). Figure 7G shows the mRNA expression of *4.1N* in TCGA database from 535 collected clinical ovarian carcinoma samples; its expression was negatively correlated with the mRNA expression of *14-3-3ε*, *14-3-3τ*, *Snail2*, *N-cadherin*, *MMP1*, *BCL2A1* and *RhoA*, as well as positively correlated with the mRNA expression of *E-cadherin*. These results suggest that the loss of 4.1N leads to the EMT, anoikis resistance, and entosis, ultimately leading to poor clinical outcomes in EOC patients.

## Discussion

As a membrane cytoskeleton cross-linker protein, 4.1N may act as a tumor suppressor by inhibiting the malignant transformation of primary cell lines, tumor growth, migration, and metastasis in various human cancers. For instance, Yang et al., indicated that by directly interacting with flotillin-1 through its FERM and U2 domains, 4.1N suppressed the β-catenin/Wnt pathway ultimately interrupting cancer cell proliferation and migration in non-small cell lung cancer (Yang et al., 2016). The pathophysiological roles of 4.1N have been further studied in non-small cell lung cancer; it has been found that low 4.1N expression activated JNK-c-Jun signaling to promote tumor occurrence and development (Wang et al., 2016). Similarly, as a tumor suppressor in breast cancers, 4.1N was also reported to be involved in repressing tumor invasiveness (Ji et al., 2012). Our previous work demonstrated the role of 4.1N in EOC patients and indicated that it can inhibit hypoxia-induced EMT in EOC cells (Xi et al., 2013; Zhang et al., 2016). However, the underlying molecular mechanisms linking the role of 4.1N in EOC progression remains to be clarified. In this study, we focused on the role of 4.1N in the entire process of EOC progression in detail and confirmed that the loss of 4.1N not only induces EMT in both adherent and suspension EOC cells, but also results in anoikis resistance and entosis-induced cell death resistance.

Many studies have reported the lethal “liaisons” between anoikis and EMT (Thiery et al., 2009), and it is thought that anoikis resistance is one of the EMT hallmarks, and EMT-like cells are more resistant to anoikis (Frisch et al., 2013; Tong et al., 2017). However, our study confirmed that EMT was an initial step in EOC, which allows cancer cells to dissociate from the basement membrane with a disordered apicobasal polarity, facilitating their disassociation from a non-cellular 3D macromolecular network in the ECM. Eventually, this leads to a mesenchymal stem cell-like phenotype for invasion and dissemination (Thiery et al., 2009; Bonnans et al., 2014). For the first time, we demonstrated the effect of 4.1N depletion on EMT led to a positive feedback loop. Specifically, 4.1N deletion exacerbates the occurrence of the EMT in adherent conditions. Cells with invasive phenotypes leave the basement membrane and become matrix-detached. In suspension, 4.1N deletion further exacerbates 14-3-3-dependent EMT by upregulating Snail transcription. Taken together, the positive feedback loop promoted EOC progression.

Integrin, which contains focal adhesion sites, plays a significant role in modulating various cellular functions, such as cell survival and movement, by transmitting ECM-derived signals across the membrane (Frisch and Francis, 1994; Giancotti, 2000). Previous studies have identified that integrin signaling is essential to induce anoikis (Brooks et al., 1994; Howlett et al., 1995; Zhang et al., 1995). Recently, a study has identified that 4.1, ezrin, radixin, moesin (FERM) domain interacts with the focal adhesion complex by binding FAK to control the catalytic activity of FAK (Frame et al., 2010). Similarly, our EOC clinical samples protein spectrum results showed that reduced 4.1N protein expression was significantly correlated with ECM and focal adhesion pathways. Furthermore, the results of our IF assays showed that the co-localization of FAK and 4.1N in suspension was significantly reduced compared to that in adherent cells (Fig. S2J). Based on our current findings, we propose the following model for the mechanism underlying the loss of integrin-mediated cell-matrix contact and the stimulation of anoikis: once integrin-mediated cell-matrix contact is dysregulated, the focal adhesion complex—which constitutively includes protein 4.1N—undergoes a loss in molecular homeostasis and changes its conformation. This is followed by potential exposure of the FERM domain of 4.1N and binding to 14-3-3.

As a highly conserved adaptor protein family, 14-3-3 proteins are known to be overexpressed in various types of cancers and may positively regulate various biological pathways that contribute to cancer progression (Hermeking, 2003; Wilker and Yaffe, 2004). Protein 14-3-3 has been reported to bind the EMT regulator Snail at its T177 residue; the mutation of this binding site is thought to lead to the deregulation of E-cadherin transcription and EMT (Hou et al., 2010). Protein 14-3-3σ was found not only to inhibit apoptosis by sequestering Bax in the cytoplasm and preventing its translocation to the mitochondria (Samuel et al., 2001), but also to negatively regulate apoptosis by changing the conformational structures of BAD to stimulate its dissociation from Bcl2/Bclxl (Tan et al., 2000). An investigation using yeast two-hybrid system screening technology reported that 14-3-3 proteins are major binding partners for 4.1N by binding the key Phe^359^ residue in the FERM domain (Calinisan et al., 2006). Our current data are consistent with the above findings. 4.1N can both promote 14-3-3 degradation and down-regulate 14-3-3-dependent Snail expression when the two are directly bound. In turn, the loss of 4.1N leads to the accumulation of 14-3-3, increased cytoplasmic sequestration, and reduced mitochondria localization of Bax. This ultimately leads to the inhibition of apoptosis.

Entosis is defined as a cell-in-cell structure formed by the invasion of one cell into another neighboring host cell. Glucose starvation (Hamann et al., 2017) and radiation/chemotherapy treatment (Martins et al., 2018) can induce entosis. Entosis is reported to play a dual role in cancer. On the one hand, entosis (termed type IV cell death) can result in cell death (Martins et al., 2017), and repeated entosis can reduce clonogenic potential. On the other hand, entosis not only acts as a mechanism of competition between malignant and benign cells, with the winner cells receiving nutrients from loser cells (Kroemer and Perfettini, 2014; Sun et al., 2014); but also induces the formation of aneuploid cells, which further accelerates the malignancy of the tumor (Janssen and Medema, 2011). In this study, we first verified the relationship between entosis and cell death. We found there is no significant effect on the cell death rate after inhibition of entosis. Co-culture of 4.1N-intact and 4.1N-deleted cells revealed that only 4.1N-deleted cells invaded 4.1N-deleted cells to form cell-in-cell structure. We further confirmed that 4.1N-deleted cells with a high entosis rate showed a marked increase in cell death resistance in matrix-detached cells. Taken together, we propose that 4.1N can negatively regulate entosis, and that 4.1N acts as a tumor suppressor, at least partially by inhibiting entosis.

In conclusion, we have shown that the loss of 4.1N was closely correlated with increased clinical stage progression, as well as poor OS and PFS in EOC patients. *In vitro*, we demonstrated that the expression of 4.1N not only down-regulated the expression of EMT-related markers and inhibited migratory and invasiveness properties in adherent EOC cells, but also inhibited anoikis resistance and EMT by directly binding and accelerating the degradation of 14-3-3 in suspended cells. Furthermore, 4.1N expression decreased the rate of entosis, which inhibits cell death resistance in suspended EOC cells. Our *in vivo* results also suggested that the loss of 4.1N could aggravate the peritoneal dissemination of EOC cells; single-agent and combination therapies with a ROCK inhibitor and a 14-3-3 antagonist successfully reduced tumor growth. Overall, our data reveal a novel mechanism for 4.1N in regulating the EMT and matrix-detachment-induced cell death resistance in EOC, and suggest that the modulation of 4.1N, as well as individual or combined treatment with 14-3-3 competitive inhibitors and entosis inhibitors, may provide a novel therapeutic approach for treating EOC.

## Materials and methods

### Patients and tissue specimens

**Figure 8:**
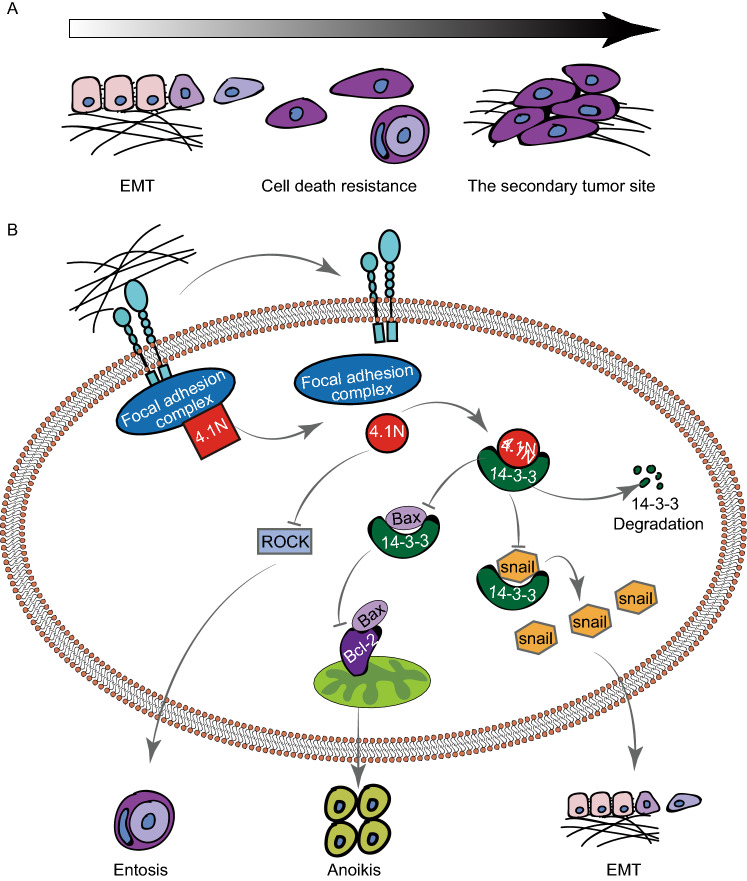
**Proposed model**. (A) Schematic model of the classical cancer progression and metastasis pathways. EMT enables cancer cells to acquire a migratory and invasive phenotype and allows them to leave the primary tumor; cell death resistance enables the survival of adherent cells in suspension; cancer cells adhere and proliferate in the new environment; the secondary tumor site can be formed. (B) 4.1N signaling in subcellular space. Under normal conditions, cells are in homeostasis. Upon the loss of integrin-mediated cell-matrix contact, the structure of the focal adhesion complex, including 4.1N, is destroyed, exposing the FERM domain of 4.1N and accelerating the degradation of 14-3-3 through binding with the FERM domain. Functionally, this decrease in 14-3-3 accumulation promotes anoikis and reduces EMT; On the other hand, the loss of 4.1N activates ROCK signaling and leads entosis induced cell-death resistance

This study was approved by the Institutional Ethics Committee of Peking University Health Science Center (approval no. IRB00001052-15064). A total of 268 [172 (64.18%) high-grade serous, 10 (3.73%) low-grade serous, 36 (13.43%) endometrioid, 37 (13.81%) clear cell, 5 (1.87%) seromucinous, 8 (2.98%) mucinous] histologically confirmed EOC samples from the Department of Pathology, School of Basic Medical Sciences, Third Hospital, Peking University Health Science Center between January 2010 and December 2017 were recruited for this study (Table S1). The end date of follow-up was the date of final contact or the date of death until September 2019. The mean follow-up period was 39.77 months (ranging from 0.07 to 114.17 months). Patient diagnoses were independently reviewed by two pathologists and classified using WHO criteria. Informed consent was obtained from all the patients in the present study (Fig. 8).

### Antibodies and reagents

Antibodies targeting the following proteins were used: anti-4.1N (donated by Dr. Xiuli An (Red Cell Physiology Laboratory, New York Blood Center, New York, USA)); 1:1,000 for Western blot (WB) and 1:100 for IHC/immunofluorescence (IF), anti-14-3-3ζ (Abcam, Cambridge, UK; Ab51129; 1:1,000 for WB, 1:1,600 for IHC, and 1:200 for IF), anti-14-3-3ζ (HuaxingBio, Beijing, China; HX12009, 5 µg/mL), anti-green fluorescent protein (GFP; Abcam; Ab290; 2 µg/mL), anti-FAK (Cell Signaling Technology, Danvers, MA, USA; #13009; 1:200), anti-phospho-myosin light chain 2 (pMLC-2; Cell Signaling Technology; #3671; 1:1,000), anti-B-cell lymphoma 2 (Bcl-2; Abcam; Ab32124; 1:1,000), anti-Bcl-2-associated death promoter (BAD; Abcam; Ab32445; 1:1,000), anti-Bcl-2-associated X (Bax; Abcam; Ab32503; 1:1,000 for WB, 1:16,000 for IHC, and 1:200 for IF), anti-Snail (Abcam; Ab53519; 1:1,000 for WB and 1:1,600 for IHC), anti-E-cadherin (Abcam; Ab40772; 1:1,000 for WB and 1:200 for IF), anti-N-cadherin (Cell Signaling Technology; #13116; 1:1,000), anti-Vimentin (Cell Signaling Technology; #5741; 1:1,000), anti-zona occludens 1 (ZO-1; Cell Signaling Technology; #8193; 1:1,000), anti-Twist (Cell Signaling Technologies; #46702; 1:1,000), anti-Slug (Cell Signaling Technology; #9585; 1:1,000 for WB), anti-zinc finger E-box-binding homeobox 1 (Zeb-1; Cell Signaling Technology; #3396; 1:1,000), anti-RhoA (Cytoskeleton Inc., Denver, CO, USA; ARH04; 1:500), anti-p190-Rho-GAP (BD Transduction Laboratories, San Jose, CA, USA; #610150; 1:1,000), and anti-β-Actin (ZSGB-Bio, Beijing, China; TA09; 1:1,000). Y27632 (40 µmol/L, 146986-50-7), MG132 (10 µmol/L, 133407-82-6), and Cycloheximide (CHX; 100 µg/mL; 66-81-9) were purchased from MedChemExpress (Monmouth Junction, NJ, USA). 14-3-3 antagonist R18 (12144) was purchased from Tocris Bioscience (Bristol, UK).

### Cell culture

The SKOV-3 ovarian cancer cell line was purchased from American Type Culture Collection (ATCC, Manassas, VA, USA), and the A2780 ovarian cancer cell line was purchased from Nanjing KeyGen Biotech (Nanjing, China). All cell lines were cultured in high-glucose Dulbecco’s modified Eagle’s medium (H-DMEM; Gibco, Thermo Fisher Scientific, Waltham, MA, USA; C11995500) supplemented with 10% fetal bovine serum (FBS; Tian Hang Biotechnologies, China), 100 U/mL penicillin, and 100 μg/mL streptomycin. All cell lines were cultured at 37 °C with 5% CO_2_.

### Transfection and lentiviral transduction

EPB41L1-pEGFP (4.1N) and negative control-pEGFP (Con) plasmids were provided by Dr. Xiuli An (Red Cell Physiology Laboratory, New York Blood Center, New York, NY, USA). EPB41L1-sgRNA-Cas9-EGFP (sg4.1N) and negative scramble control-EGFP (sgCon) lentiviral plasmids were purchased from GeneChem (Shanghai, China). The following single-guide RNAs (sgRNA) and primers were used to generate the knockout plasmids: 4.1N-knockout SKOV3 human sgRNA forward, sgRNA-1: 5′-GAGGGCATCGACATCATGTT-3′, sgRNA-2: 5′-CAGTAGGGCATGCGTGACAA-3′, sgRNA-3: 5′-TCCTCAAGATCTCCTACAAG-3′. A single-cell clone from the lentivirus-infected cell pool was selected and verified by WB and DNA sequencing. EPB41L1-pGEX-4T-1 (GST-4.1N) and negative control (GST) plasmids were purchased from MailGene (Beijing, China). YWHAZ-GFP (14-3-3ζ), YWHAZ-RNAi (14-3-3ζ shRNA), and relative negative control plasmids were purchased from GeneChem. Lentivirus infection was performed with polybrene (GeneChem, REVG0001), enhanced infection solution (ENi.S.; GeneChem, REVG0002), and Polyplus transfection reagent (PolyPlus Transfection, Illkirch-Graffenstaden, France; jetPRIME) according to the manufacturer’s instructions.

### Anoikis assay

First, 6-well or 96-well plates were coated with poly-HEMA (2-hydroxyethyl methacrylate; 20 mg/mL in absolute ethanol) to prevent cells from adhering to the base of culture dishes. This was performed to mimic the anchorage-independent growth conditions of the cells. Cells were plated in DMEM and collected by centrifugation after incubation for 24 h.

### Quantification of entosis structures and winner/loser cell identity

To quantify cell-in-cell structures, cells were cultured in suspension for 6 h on poly HEMA-coated plates and then mounted onto glass slides by Cytospin preparation and fixation in 10% trichloroacetic acid (TCA). Cells were immunostained with E-cadherin antibodies for 12 h, Cy3-AffiniPure goat anti-rabbit secondary antibodies (Yeasen Biotech Co., Shanghai, China) for 2 h, and DAPI (Beijing Solarbio Science & Technology Co., Beijing, China), and then imaged by confocal microscopy. Cell structures showing internalization of more than half of the cell body were counted.

For winner/loser identity analysis, equal concentrations of cells from two cell lines were labeled with CellTracker Green CMFDA (Yeasen Biotech Co., Ltd, 40721ES50) and CellTracker Red CM-Dil (Yeasen Biotech Co., Ltd, 40718ES50) and co-cultured in suspension. The cells were then plated onto glass slides for analysis by confocal microscopy.

### IHC

IHC and semi-quantitative scoring were performed as described previously (Xi et al., 2013). According to their total scores, they were divided into two groups for each protein: low expression of 4.1N (4.1N^-^): 0–3 points, and high expression of 4.1N (4.1N^+^): 4–9 points; low expression of 14-3-3ζ (14-3-3ζ^-^): 0–5 points, high expression of 14-3-3ζ (14-3-3ζ^+^): 6–9 points; low expression of Bax (Bax^-^): 0–2 points, high expression of Bax (Bax^+^): 3–9 points; low expression of Snail (Snail^-^): 0–3 points, and high expression of Snail (Snail^+^): 4–9 points.

### IF

Adherent cells on glass coverslips (NEST Biotechnology, Wuxi, China) or cell suspensions collected by centrifugation were fixed with 4% paraformaldehyde (PFA) and permeabilized with 0.1% Triton X-100 in phosphate-buffered saline (PBS). Samples were blocked in 5% goat serum, stained with the appropriate fluorescence-coupled primary and secondary antibodies, and imaged by confocal microscopy (TCS SP2AOBS, Leica Microsystems, Wetzlar, Germany).

### WB analysis

WB was performed as described previously (Zhang et al., 2016). Cells were lysed in radioimmune precipitation assay buffer (Applygen Technologies, Beijing, China; C1053) mixed with protease and phosphatase inhibitors (Thermo Fisher Scientific, 87786; Applygen Technologies, P1265, P1260). The cells were then centrifuged at 12,000 ×*g* for 10 min at 4 °C and supernatant was collected. Proteins were denatured in sodium dodecyl sulfate (SDS) sample buffer (Applygen Technologies, B1012) at 100 °C for 5 min and separated by electrophoresis on a 10% or 15% SDS‑polyacrylamide gel electrophoresis (Applygen Technologies), then transferred to a nitrocellulose membrane (Applygen Technologies). Membranes were incubated in blocking buffer (5% non‑fat milk, 0.1% Tween 20 in Tris-buffered saline (TBST)) for 2 h at 37 °C and probed overnight with primary antibody at 4 °C. The membranes were washed thrice in TBST and incubated with peroxidase-conjugated secondary antibody (Beijing Zhongshan Golden Bridge Biotechnology, Beijing, China) for 2 h at room temperature. Proteins were detected using Super Enhanced Chemiluminescence Detection Kit (Applygen Technologies). The Gel Doc XR+ imaging system (Bio-Rad Laboratories, Hercules, CA, USA) was used to capture images.

### Quantitative reverse transcriptase PCR

Quantitative reverse transcriptase PCR (qRT‑PCR) was performed as previously described (Zhang et al., 2016). The primer sequences used for qRT-PCR are listed in Table S2. Gene expression levels were normalized to β-Actin. The relative expression level of mRNA was evaluated by the 2^−ΔΔCt^ method.

### Live cell imaging

Live cell imaging was performed using total internal reflection fluorescent microscopy (TIRF; Olympus, Tokyo, Japan). Cells were plated on 96-well plates coated with poly-HEMA and maintained in DMEM supplemented with 10% FBS, 100 U/mL penicillin, and 100 µg/mL streptomycin at 37 °C and 5% CO_2_ throughout the imaging process. Cells were imaged every 10 min for 24 h.

### Co-immunoprecipitation assay and co-immunoprecipitation/liquid chromatography-tandem mass spectrometry for 4.1N

For co-immunoprecipitation (Co-IP), A2780-WT and A2780-OE cells were plated on 10 cm dishes and cell lysates were prepared by incubating the cells in lysis buffer (50 mmol/L Tris-HCl, pH 8.0, 150 mmol/L NaCl, 0.2% Nonidet P-40, 2 mmol/L EDTA) in the presence of protease and phosphatase inhibitors (Thermo Fisher Scientific, Applygen Technologies; P1265, P1260) for 20 min at 4 °C. Next, 2 µg anti-GFP antibody was added to the lysate and incubated at 4 °C overnight, and 50 µL 50% protein A/G agarose beads (Santa Cruz Biotechnology, Santa Cruz, CA, USA) were added to the lysate and incubated for 4 h at 4 °C. The beads were washed with lysis buffer five times and centrifuged at 12,000 ×*g* for 10 min at 4 °C. Precipitated proteins were eluted from the beads by re-suspending them in 2× SDS-PAGE loading buffer and boiling for 5 min. Boiled immune complexes were subjected to SDS-PAGE, followed by immunoblotting with the appropriate antibodies.

Mass spectrometry is a powerful tool to screen and identify new interacting proteins from Co-IP products. After a proof-of-concept confirmation of on-bead crosslinking using Western blot, Co-IP/liquid chromatography-tandem mass spectrometry (LC-MS/MS) analysis was performed for 4.1N. Thus, 225 proteins were identified in the A2780 OE group, and 212 proteins were identified in the A2780 WT group. Only 38 proteins in the experimental group were found to interact with 4.1N.

### GST (glutathione S-transferase) pull-down assay

GST pull-down assays were performed using a GST Protein Interaction Pull-Down Kit (Pierce Biotechnologies, Waltham, MA, USA). GST fusion protein was produced in *Escherichia coli* strains for 4 h at 37 °C after expression was induced with 0.1 mmol/L IPTG (isopropyl β- d-1-thiogalactopyranoside; Sigma Aldrich, St. Louis, MO, USA). Finally, protein complexes were visualized by WB.

### Migration and invasion assays

For the wound-healing migration assay, cells were seeded in 6-well plates for 24 h, and then a 200 μL pipette tip was used to scratch the cells. The cells were then washed with PBS and cultured in FBS-free DMEM for 18 h. Wounds were observed under a microscope and photographed at 0, 6, 12, and 18 h.

The transwell migration assay was performed using BD BioCoat Matrigel Invasion Chambers (8 μm pore size; BD Biosciences, San Jose, CA, USA). Next, 600 μL DMEM containing 10% FBS was added to the lower chamber, and 10^4^ (10,000) cells/100 μL serum-free media were placed into the upper chamber of the transwell insert. After incubation for 24 h, cells remaining on the upper membrane were removed with a cotton swab, while cells that had invaded through the membrane were fixed in formaldehyde, stained with crystal violet, and counted using an Olympus fluorescence microscope.

The filters were pre-coated with 100 μL Matrigel at 1:4 dilution in DMEM to form a genuine reconstituted basement, then 600 μL DMEM containing 10% FBS was added to the lower chamber, and 10^4^ cells/100 μL serum-free media were placed into the upper chamber of the transwell insert. After incubation for 24 h, cells remaining on the upper membrane were removed with a cotton swab, while cells that had invaded through the membrane were fixed in formaldehyde, stained with crystal violet, and counted using an Olympus fluorescence microscope.

### Real-time invasion assay

The invasion curve assay was performed using a real-time cell analyzer (RTCA) with the xCELLigence system CIM-plates (ACEA Bioscience, San Diego, CA, USA) according to the manufacturer’s instructions. The upper chamber of the CIM-plates was coated with 100 μL Matrigel at 1:4 dilution in DMEM to form a genuine reconstituted basement. A total of 100 cells/μL were seeded in each well of the upper chamber in serum-free DMEM, and 165 μL DMEM containing 10% FBS was added to the lower chamber. The CIM-plates were incubated in an incubator for 1 h. The impedance value of each well was automatically monitored by the xCELLigence system over 48 h and expressed as a cell index (CI) value.

### Flow cytometry for detection of apoptosis

To analyze the apoptosis rate, cells were collected by centrifugation at 300 ×*g* for 5 min at 4 °C. The cells were then washed twice with PBS precooled to 4 °C, followed by the addition of 250 µL 1× binding buffer to re-suspend and adjust the cells to 1 × 10^6^ cells/mL. Cells were stained with the Annexin V-PE/7-AAD Apoptosis Detection Kit (Yeasen Biotech Co.) and Annexin V-Alexa Fluor 647 Apoptosis Detection Kit (Bioss Antibodies, Beijing, China; BA00103) and analyzed with a flow cytometer.

### Assessment of mitochondria dysfunction by JC-1 staining

Mitochondrial potential dysfunction was assessed by staining with JC-1 (Yeasen Biotech Co., Ltd., 40706ES60–100-kit), a sensitive fluorescent dye used to detect changes in mitochondrial potential. JC-1 is a red fluorescent dye that forms aggregates in healthy or normal mitochondria. However, JC-1 monomers emit green fluorescence in cells with injured or damaged mitochondrial membranes. Cells were seeded in 24-well plates for 24 h. Next, the medium was discarded and replaced with 500 μL fresh medium containing 1X JC-1. After treatment, cells were washed three times in PBS and then observed under a confocal microscope (TCS SP2AOBS, Leica Microsystems). ImageJ software was used to obtain the mean densities of the region of interest and the red to green fluorescence ratio to quantify the extent of mitochondrial membrane depolarization.

### Trypan blue staining

To observe the rates of cell death, trypan blue staining was used. Cell were treated with 0.4% trypan blue (Beijing Solarbio Science & Technology Co., Ltd.; C0040-50 mL) for 3–5 min, and the number of trypan blue-positive cells was calculated by counting at least three random separate fields.

### *In vivo* xenograft mouse model

Animal studies were conducted according to guidelines approved by the Biomedical Ethics Committee of Peking University (approval no. LA2014136). Female BALB/c nude (5-week-old) were maintained in specific pathogen-free conditions at the Center of Experimental Animals (Peking University, Beijing, China). Sixty-four animals were randomly distributed into eight groups. A2780 OE (2 × 10^6^) and A2780 WT cells (2 × 10^6^) were injected into the left flank and right flank, respectively. Then, 5 × 10^6^ A2780 OE or A2780 WT cells were injected into the peritoneal cavity. Mice were treated with single-agent or combined therapies by injection using the ROCK inhibitor Y27632 and/or the 14-3-3 antagonist R18 after three weeks. Animals were then monitored for tumor growth. After six weeks, tumors were detected using IVIS Imaging System (Caliper Life Sciences, Waltham, MA, USA), and animals were sacrificed and photographed. Tumors from the abdominal area were collected, weighed, photographed, and snap-frozen in liquid nitrogen or formalin-fixed and paraffin-embedded. Paraffin-embedded tumor tissues underwent routine histological processing with hematoxylin and eosin (H&E) staining. All protocols in this study were performed in accordance with the Declaration of Helsinki.

### Statistical analysis

Data were assessed using SPSS version 20.0 (SPSS Inc., Chicago, IL, USA). Adobe Illustrator CC 2018 and GraphPad Prism 5.0 (GraphPad Software Inc., San Diego, CA, USA) were used to represent the data. Expression and functional data were analyzed using Student’s *t*-test or analysis of variance (ANOVA), followed by the Bonferroni post-hoc test. To verify correlation, the Spearman correlation coefficient was used. All data are represented as the mean ± standard error of mean (SEM). *P* < 0.05 was considered to indicate a statistically significant difference.

## Electronic supplementary material

Below is the link to the electronic supplementary material.

**Supplementary figure 1.** Loss of 4.1N exacerbates EOC aggressiveness. (A) Representative images of the low expression and high expression of 4.1N in EOC. Scale bar, 200μm (left) and 50μm (right). (B) Representative images of the low expression and high expression of 14-3-3ζ, Bax and Snail in EOC. Scale bar, 100μm. (C and D) The Kaplan-Meier plot showed the Progression-free survival in comparison of patients with high or low 4.1N expression in our data and TCGA database (*p* = 3.53E-10 and 0.048, respectively). (E and F) qRT-PCR and western blot assay of 4.1N in SKOV3 single clones after using specific 4.1N sgRNAs by CRISPR, as well as after transfected 4.1N-overexpressed plasmid in A2780. β-actin was used as internal control. (G) Representative images of nude mice formed by indicated treatment. Body weight (H) and percentage of survival (I) curve in nude mice.Additional file 2: **Supplementary figure 2.** Loss of 4.1N promotes EOC cells EMT, anoikis resistance and entosis-cell death resistance *in vitro*. (A) SKOV3 KO cells treated with Y27632 at the 0, 10, 20, 40, 80μM concentrations for 24 h. The formation ratio of entosis was measured. (B) A2780 WT, A2780 OE, SKOV3 WT and SKOV3 KO cells (2000 cells/96 well) in suspension treated Y27632 at 40μM, cell viability measured by CCK8 at 0, 24, 48, 72, 96, 120h and presented as means ± SD from three separate experiments. (C and D) 4.1N and 14-3-3ζ mRNA expression was assayed by qRT-PCR and results means ± SD from three independent experiments. β-actin was used as internal control. (E) The proliferative ability of adherent SKOV3 cells at different fetal bovine serum (FBS) concentrations in 48h. (F) Representative images of flow cytometry results with 7AAD–Annexin V-AF647 staining, A2780 WT, A2780 OE, SKOV3 WT and SKOV3 KO cells in adherent. (G and H) Representative images of flow cytometry results with 7AAD–Annexin V-PE staining, A2780 WT, A2780 OE, SKOV3 WT and SKOV3 KO cells in suspension, results means ± SD from three independent experiments. (I) The trypan blue assay was used to detect the cellular death. (J) Immunoprecipitation assay to detect subcellular localization of 4.1N and FAK. Scale bar, 10 µm. *p < 0.05, ** *p* < 0.01 and *** *p* < 0.001 (Student’s t test).Additional file 3: **Supplementary figure 3.** 4.1N negatively regulates 14-3-3 expression. (A) Protein-protein network predicting highly potential interactions with 4.1N genes based on BioGrid databases. (B) The relevance of 4.1N and 14-3-3 mRNA expression from Gene Expression Omnibus (GEO) dataset (GSE21501, containing samples from 527 EOC patients) by R2 platform (http://r2.amc.nl). (PDF 116800 kb)

Supplementary Movie (AVI 110 kb)
