# Supplementary material for: Loss of 4.1N in epithelial ovarian cancer results in EMT and matrix-detached cell death resistance
Source: Protein Cell. 2020 May 25;12(2):107–27. doi: 10.1007/s13238-020-00723-9 (PMC7862473; doi:10.1007/s13238-020-00723-9)
Supplement: Supplementary file 1 — Supplementary figure 1. Loss of 4.1N exacerbates EOC aggressiveness. (A) Representative images of the low expression and high expression of 4.1N in EOC. Scale bar, 200μm (left) and 50μm (right). (B) Representative images of the low expression and high expression of 14-3-3ζ, Bax and Snail in EOC. Scale bar, 100μm. (C and D) The Kaplan-Meier plot showed the Progression-free survival in comparison of patients with high or low 4.1N expression in our data and TCGA database (p = 3.53E-10 and 0.048, respectively). (E and F) qRT-PCR and western blot assay of 4.1N in SKOV3 single clones after using specific 4.1N sgRNAs by CRISPR, as well as after transfected 4.1N-overexpressed plasmid in A2780. β-actin was used as internal control. (G) Representative images of nude mice formed by indicated treatment. Body weight (H) and percentage of survival (I) curve in nude mice.Additional file 2: Supplementary figure 2. Loss of 4.1N promotes EOC cells EMT, anoikis resistance and entosis-cell death resistance in vitro. (A) SKOV3 KO cells treated with Y27632 at the 0, 10, 20, 40, 80μM concentrations for 24 h. The formation ratio of entosis was measured. (B) A2780 WT, A2780 OE, SKOV3 WT and SKOV3 KO cells (2000 cells/96 well) in suspension treated Y27632 at 40μM, cell viability measured by CCK8 at 0, 24, 48, 72, 96, 120h and presented as means ± SD from three separate experiments. (C and D) 4.1N and 14-3-3ζ mRNA expression was assayed by qRT-PCR and results means ± SD from three independent experiments. β-actin was used as internal control. (E) The proliferative ability of adherent SKOV3 cells at different fetal bovine serum (FBS) concentrations in 48h. (F) Representative images of flow cytometry results with 7AAD–Annexin V-AF647 staining, A2780 WT, A2780 OE, SKOV3 WT and SKOV3 KO cells in adherent. (G and H) Representative images of flow cytometry results with 7AAD–Annexin V-PE staining, A2780 WT, A2780 OE, SKOV3 WT and SKOV3 KO cells in suspension, results means ± SD from three independen [file 13238_2020_723_MOESM1_ESM.pdf]

Supplementary Table 1. Association between 4.1N expression and clinicopathological characteristics in EOC

		4.1N expression		
		Low expression	High expression	
Characteristics	n=268(%)	n=168(%)	n=100(%)	*p* value
Age (years)				0.093
<50	103(38.4)	58(34.5)	45(45.0)	
≥50	165(61.6)	110(65.5)	55(55.0)	
FIGO stage				0.008**
I	65(24.25%)	33(19.6)	32(32.0)	
II	31(11.57%)	16(9.5)	15(15.0)	
III	138(51.49%)	91(54.2)	47(47.0)	
IV	34(12.69%)	28(16.7)	6(6.0)	
Tumor histology				0.002**
High-grade serous	172(64.18%)	121(72.0)	51(51.0)	
Low-grade serous	10(3.73%)	6(3.6)	4(4.0)	
Clear cell	37(13.81%)	18(10.7)	19(19.0)	
Endometrioid	36(13.43%)	14(8.3)	22(22.0)	
Mucinous	8(2.98%)	7(4.2)	1(1.0)	
Seromucino	5(1.87%)	2(1.2)	3(3.0)	

**p<0.01 represents the p-values with significant differences.

Supplementary Table 2. Primer sequences for qRT-PCR

Gene	Primer
4.1N	Forward: 5'-AGGAAACCACGCCGAGACACAA-3'
	Reverse: 5'-GGTGGATGAGTTTGCTGTTGGG-3'
E-cadherin	Forward: 5'-ATTTTTCCCTCGACACCCGAT-3'
	Reverse: 5'-TCCCAGGCGTAGACCAAGA-3'
N-cadherin	Forward: 5'-ACAGTGGCCACCTACAAAGG-3'
	Reverse: 5'-CCGAGATGGGGTTGATAATG-3'
ZO-1	Forward: 5'-TATTATGGCACATCAGCACG-3'
	Reverse: 5'-TGGGCAAACAGACCAAGC-3'
Fibronectin	Forward: 5'-TCCCTCGGAACATCAGAAAC-3'
	Reverse: 5'-CAGTGGGAGACCTCGAGAAG-3'
Snail	Forward: 5'-TCGGAAGCCTAACTACAGCG-3'
	Reverse: 5'-GATGAGCATTGGCAGCGAG-3'
MMP2	Forward: 5'-TTTCCATTCCGCTTCCAGGGCAC-3'
	Reverse: 5'-TCGCACACCACATCTTTCCGTCACT-3'
Bax	Forward: 5'-CTAAAGTGCCCGAGCTGAGCA-3'
	Reverse: 5'-TGAGGACTCCAGCCACAAAGA-3'
Bcl-2	Forward: 5'-ATAACCGGGAGATCGTGATG-3'
	Reverse: 5'-CAGGCTGGAAGGAGAAGATG-3'
14-3-3ζ	Forward: 5'- CCTGCATGAAGTCTGTAACTGAG-3'
	Reverse: 5'- GACCTACGGGCTCCTACAACA-3'
β-actin	Forward: 5'-AGCACAGAGCCTCGCCTTTG-3'
	Reverse: 5'-ACATGCCGGAGCCGTTGT-3'
GAPDH	Forward: 5'-AAGGTCGGAGTCAACGGATT-3'
	Reverse: 5'-CCATGGGTGGAATCATATTGG-3'

Supplementary Table 3. Interaction proteins of 4.1N

Accession	PROTEIN	GENE	Score	Coverage
Q9H4G0	4.1N	EPB41L1	185.15	40.41
P06733	Alpha-enolase	ENO1	44.5	43.09
Q00610	Clathrin heavy chain 1	CLTC	44.45	15.76
P68371	Tubulin beta-4B chain	TUBB4B	26.49	34.16
P60842	Eukaryotic initiation factor 4A-I	EIF4A1	22.84	20.94
P14625	Endoplasmin	HSP90B1	20.12	12.08
P12956	X-ray repair cross-complementing protein 6	XRCC6	17.9	14.45
P17844	Probable ATP-dependent RNA helicase DDX5	DDX5	16.59	11.89
P10412	Histone H1.4	HIST1H1E	14.14	23.29
P14866	Heterogeneous nuclear ribonucleoprotein L	HNRNPL	14.09	14.09
Q9NZI8	Insulin-like growth factor 2 mRNA-binding protein 1	IGF2BP1	12.51	14.9
P50990	T-complex protein 1 subunit theta	CCT8	12.05	11.31
P27797	Calreticulin	CALR	11.49	11.75
Q9UGI8	Testin	TES	11.22	13.3
P23284	Peptidyl-prolyl cis-trans isomerase B	PPIB	10.93	22.69
P26641	Elongation factor 1-gamma	EEF1G	10.61	11.21
P69905	Hemoglobin subunit alpha	HBA1	10.07	19.01
P02042	Hemoglobin subunit delta	HBD	9.8	21.77
P62937	Peptidyl-prolyl cis-trans isomerase A	PPIA	8.32	18.79
Q15366	Poly(rC)-binding protein 2	PCBP2	7.58	10.41
P62917	60S ribosomal protein L8	RPL8	6.87	13.62
Q99623	Prohibitin-2	PHB2	6.85	10.03
P21796	Voltage-dependent anion-selective channel protein 1	VDAC1	6.82	10.25
Q9H0U4	Ras-related protein Rab-1B	RAB1B	6.76	14.93
P62266	40S ribosomal protein S23	RPS23	6.68	15.38
P63104	14-3-3 zeta/delta	YWHAZ	5.95	22.45
P61981	14-3-3 gamma	YWHAG	5.63	12.96
Q13151	Heterogeneous nuclear ribonucleoprotein A0	HNRNPA0	5.27	13.77
P60866	40S ribosomal protein S20	RPS20	5.24	15.13
Q04917	14-3-3 eta	YWHAH	4.93	13.01
P23528	Cofilin-1	CFL1	4.63	20.48
P09211	Glutathione S-transferase P	GSTP1	4.3	14.76
P63241	Eukaryotic translation initiation factor 5A-1	EIF5A	4.09	20.13
P35080	Profilin-2	PFN2	3.66	10
P62913	60S ribosomal protein L11	RPL11	3.15	11.8
P62244	40S ribosomal protein S15a	RPS15A	2.25	10.77
P63220	40S ribosomal protein S21	RPS21	2.03	12.05
Q9H3K6	BolA-like protein 2	BOLA2	0	18.6

Supplementary Table 4. 89 differential proteins in four clinical EOC samples.

GeneID	log2 Ratio	Pvalue
RAB5B Ras-related protein Rab-5B	0.09	0.03
S100A9 Protein S100-A9	0.09	0.00
S100A9 Protein S100-A9	0.09	0.00
BANF1 Barrier-to-autointegration factor	0.10	0.01
HBA2;HBA1 Hemoglobin subunit alpha	0.11	0.00
ALB Isoform 1 of Serum albumin	0.11	0.00
VTN Vitronectin	0.12	0.00
FGG Uncharacterized protein	0.13	0.00
CRABP1 Cellular retinoic acid-binding protein 1	0.14	0.05
FGA Isoform 1 of Fibrinogen alpha chain	0.14	0.00
SPRR3 Uncharacterized protein	0.15	0.00
FGB Fibrinogen beta chain	0.16	0.00
MPO Isoform H17 of Myeloperoxidase	0.17	0.00
GSTA1 Glutathione S-transferase A1	0.18	0.00
AZU1 Azurocidin	0.19	0.00
SRSF3 Serine/arginine-rich splicing factor 3	0.20	0.02
SERPINA1 Isoform 1 of Alpha-1-antitrypsin	0.21	0.00
DEFA1;DEFA1B Neutrophil defensin 1	0.22	0.04
C1orf31 cDNA FLJ60021, moderately similar to Protein transport protein Sec61 subunit alpha isoform 1	0.23	0.04
HBB Hemoglobin subunit beta	0.23	0.00
ORM1 Alpha-1-acid glycoprotein 1	0.24	0.01
CLU Isoform 5 of Clusterin	0.25	0.00
KRT5 Keratin, type II cytoskeletal 5	0.25	0.00
KRT5 Keratin, type II cytoskeletal 5	0.25	0.00
C9 Complement component C9	0.25	0.00
S100A8 Protein S100-A8	0.26	0.00
LTF Uncharacterized protein	0.26	0.00
HP;HPR Haptoglobin	0.26	0.00
NPC2 cDNA FLJ59142, highly similar to Epididymal secretory protein E1	0.26	0.00
SERPINF2 Alpha-2-antiplasmin	0.28	0.05
MAPRE1 cDNA FLJ52068, highly similar to Microtubule-associated protein RP/EB family member 1	0.29	0.02
APOE Apolipoprotein E	0.30	0.00
AHSG cDNA FLJ55606, highly similar to Alpha-2-HS-glycoprotein	0.30	0.01
SLC4A1 Solute carrier family 4, anion exchanger, member 1	0.32	0.01
FTL Ferritin light chain	0.32	0.01
APOC2 Apolipoprotein C-II	0.34	0.01
CTSG Cathepsin G	0.35	0.00
IGHV4-31;IGHG1 Putative uncharacterized protein DKFZp686G11190	0.35	0.03
LCN2 Neutrophil gelatinase-associated lipocalin	0.37	0.05
TXN Thioredoxin	0.38	0.00

Supplementary Table 4. 89 differential proteins in four clinical EOC samples.

GeneID	log2 Ratio	Pvalue
PLG Plasminogen	0.38	0.00
GPX1 Glutathione peroxidase 1	0.39	0.04
LGALS3BP Galectin-3-binding protein	0.39	0.01
SRSF9 Serine/arginine-rich splicing factor 9	0.41	0.00
HBD Hemoglobin subunit delta	0.42	0.03
RPL18A 60S ribosomal protein L18a	0.42	0.00
LYZ Lysozyme C	0.43	0.01
HINT1 Histidine triad nucleotide-binding protein 1	0.44	0.04
RPS17;RPS17L 40S ribosomal protein S17	0.45	0.01
TCEB2 Transcription elongation factor B polypeptide 2	0.46	0.01
RPLP0 60S acidic ribosomal protein P0	0.46	0.02
UCHL1 Ubiquitin carboxyl-terminal hydrolase isozyme L1	0.46	0.04
APOA1 Apolipoprotein A-I	0.46	0.00
PKM2 Isoform M2 of Pyruvate kinase isozymes M1/M2	0.47	0.00
- Transthyretin	0.48	0.01
TF Serotransferrin	0.49	0.04
SOD1 Superoxide dismutase [Cu-Zn]	0.50	0.05
GAPDH Glyceraldehyde-3-phosphate dehydrogenase	0.51	0.02
TRMT112 tRNA methyltransferase 112 homolog	0.52	0.03
RPL35A 60S ribosomal protein L35a	0.52	0.01
DHX15 Uncharacterized protein	0.54	0.02
PRKCSH glucosidase 2 subunit beta isoform 2	0.55	0.03
RBMX Heterogeneous nuclear ribonucleoprotein G	0.55	0.00
FN1 Isoform 15 of Fibronectin	0.56	0.00
STMN1 Isoform 1 of Stathmin	0.58	0.04
C3 Complement C3 (Fragment)	0.61	0.00
A1BG Isoform 1 of Alpha-1B-glycoprotein	0.61	0.03
COL6A1 Collagen alpha-1(VI) chain	1.66	0.01
COL1A1 Collagen alpha-1(I) chain	1.71	0.00
THBS1 Thrombospondin-1	1.74	0.01
AHNAK Neuroblast differentiation-associated protein AHNAK	1.80	0.00
LMNA Isoform A of Prelamin-A/C	1.82	0.01
DES Desmin	1.87	0.03
DCN Isoform A of Decorin	1.94	0.01
COL6A3 Isoform 1 of Collagen alpha-3(VI) chain	2.00	0.00
OGN cDNA FLJ59205, highly similar to Mimecan	2.15	0.02
COL6A2 Isoform 2C2 of Collagen alpha-2(VI) chain	2.25	0.01
MZB1 Isoform 1 of Plasma cell-induced resident endoplasmic reticulum protein	2.25	0.01
COL14A1 Isoform 2 of Collagen alpha-1(XIV) chain	2.40	0.00
PSMB8 30 kDa protein	2.51	0.04

Supplementary Table 4. 89 differential proteins in four clinical EOC samples.

GeneID	log2 Ratio	Pvalue
KRT7 Keratin, type II cytoskeletal 7	2.65	0.00
KRT10 Keratin, type I cytoskeletal 10	2.70	0.00
S100A6 Protein S100-A6	2.70	0.02
COL1A2 Collagen alpha-2(I) chain	3.02	0.00
ANXA4 annexin A4	3.05	0.00
KRT2 Keratin, type II cytoskeletal 2 epidermal	3.05	0.00
KRT9 Keratin, type I cytoskeletal 9	3.25	0.00
KRT1 Keratin, type II cytoskeletal 1	3.40	0.00
MTDH Uncharacterized protein	3.87	0.05

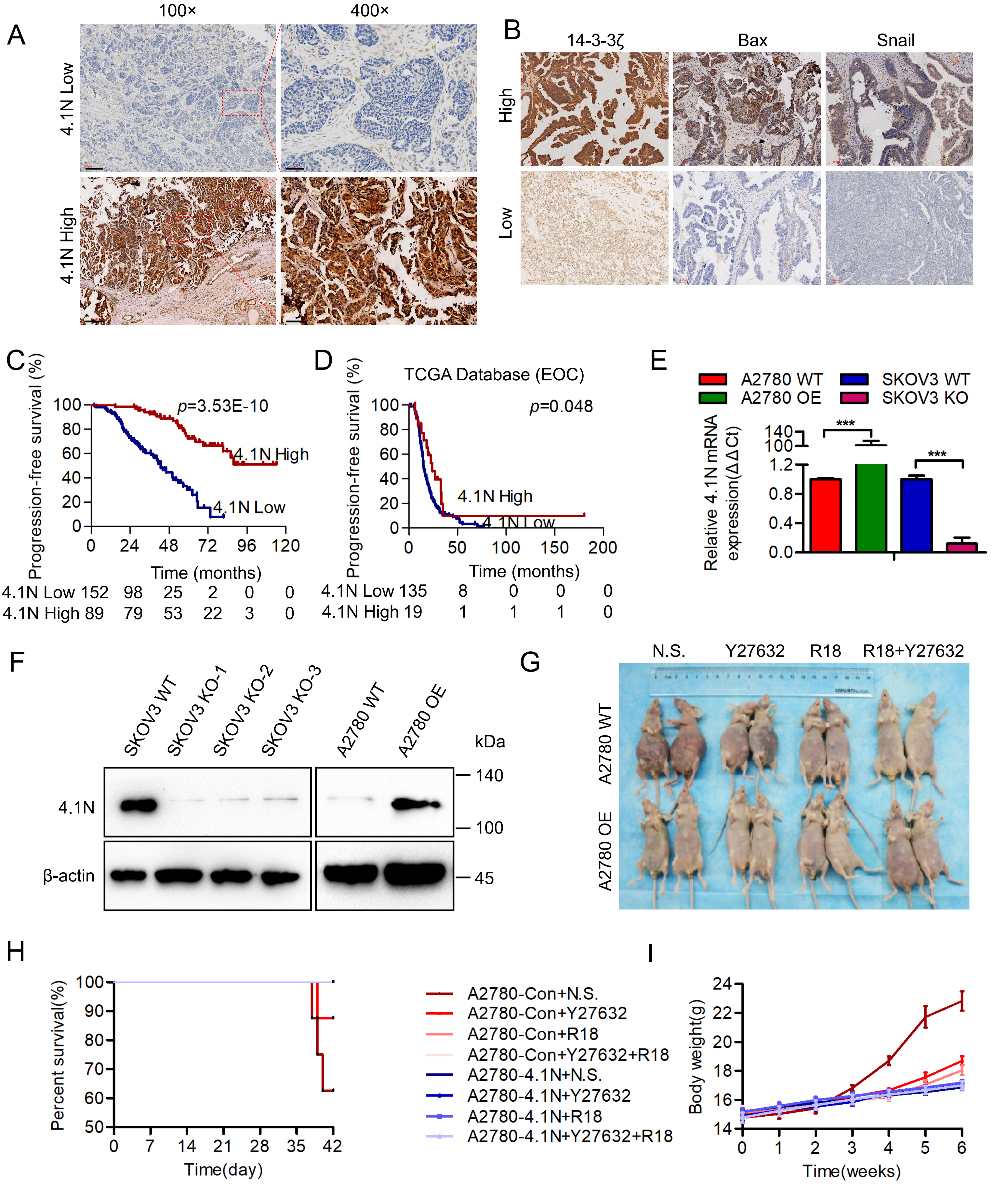

A
100×
400×
4.1N Low
4.1N High
B
14-3-3ζ
Bax
Snail
High
Low
C
p=3.53E-10
4.1N High
4.1N Low
Progression-free survival (%)
Time (months)
4.1N Low 152 98 25 2 0 0
4.1N High 89 79 53 22 3 0
D
TCGA Database (EOC)
p=0.048
4.1N High
4.1N Low
Progression-free survival (%)
Time (months)
4.1N Low 135 8 0 0 0
4.1N High 19 1 1 1 0
E
A2780 WT
A2780 OE
SKOV3 WT
SKOV3 KO
Relative 4.1N mRNA expression(ΔΔCt)
***
***
F
SKOV3 WT
SKOV3 KO-1
SKOV3 KO-2
SKOV3 KO-3
A2780 WT
A2780 OE
kDa
4.1N
β-actin
140
100
45
G
N.S.
Y27632
R18
R18+Y27632
A2780 WT
A2780 OE
H
Percent survival(%)
Time(day)
A2780-Con+N.S.
A2780-Con+Y27632
A2780-Con+R18
A2780-Con+Y27632+R18
A2780-4.1N+N.S.
A2780-4.1N+Y27632
A2780-4.1N+R18
A2780-4.1N+Y27632+R18
I
Body weight(g)
Time(weeks)

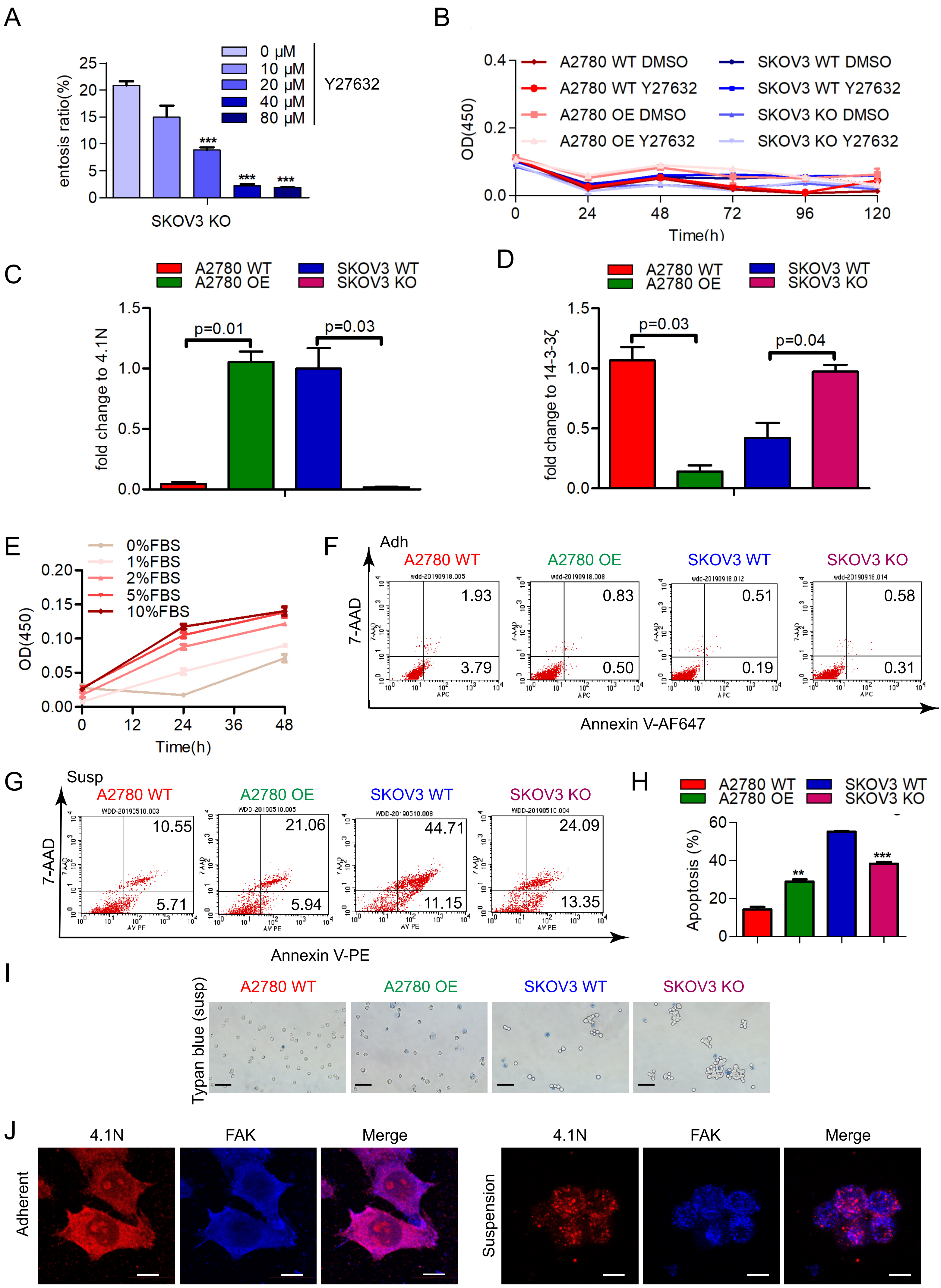

A
entosis ratio(%)
0 μM
10 μM
20 μM
40 μM
80 μM
Y27632
***
SKOV3 KO
B
OD(450)
A2780 WT DMSO
A2780 WT Y27632
A2780 OE DMSO
A2780 OE Y27632
SKOV3 WT DMSO
SKOV3 WT Y27632
SKOV3 KO DMSO
SKOV3 KO Y27632
Time(h)
C
A2780 WT
A2780 OE
SKOV3 WT
SKOV3 KO
fold change to 4.1N
p=0.01
p=0.03
D
fold change to 14-3-3ζ
p=0.03
p=0.04
E
0%FBS
1%FBS
2%FBS
5%FBS
10%FBS
OD(450)
Time(h)
F
Adh
7-AAD
1.93
3.79
0.83
0.50
0.51
0.19
0.58
0.31
Annexin V-AF647
G
Susp
10.55
5.71
21.06
5.94
44.71
11.15
24.09
13.35
Annexin V-PE
H
Apoptosis (%)
**
***
I
Typan blue (susp)
J
4.1N
FAK
Merge
Adherent
Suspension

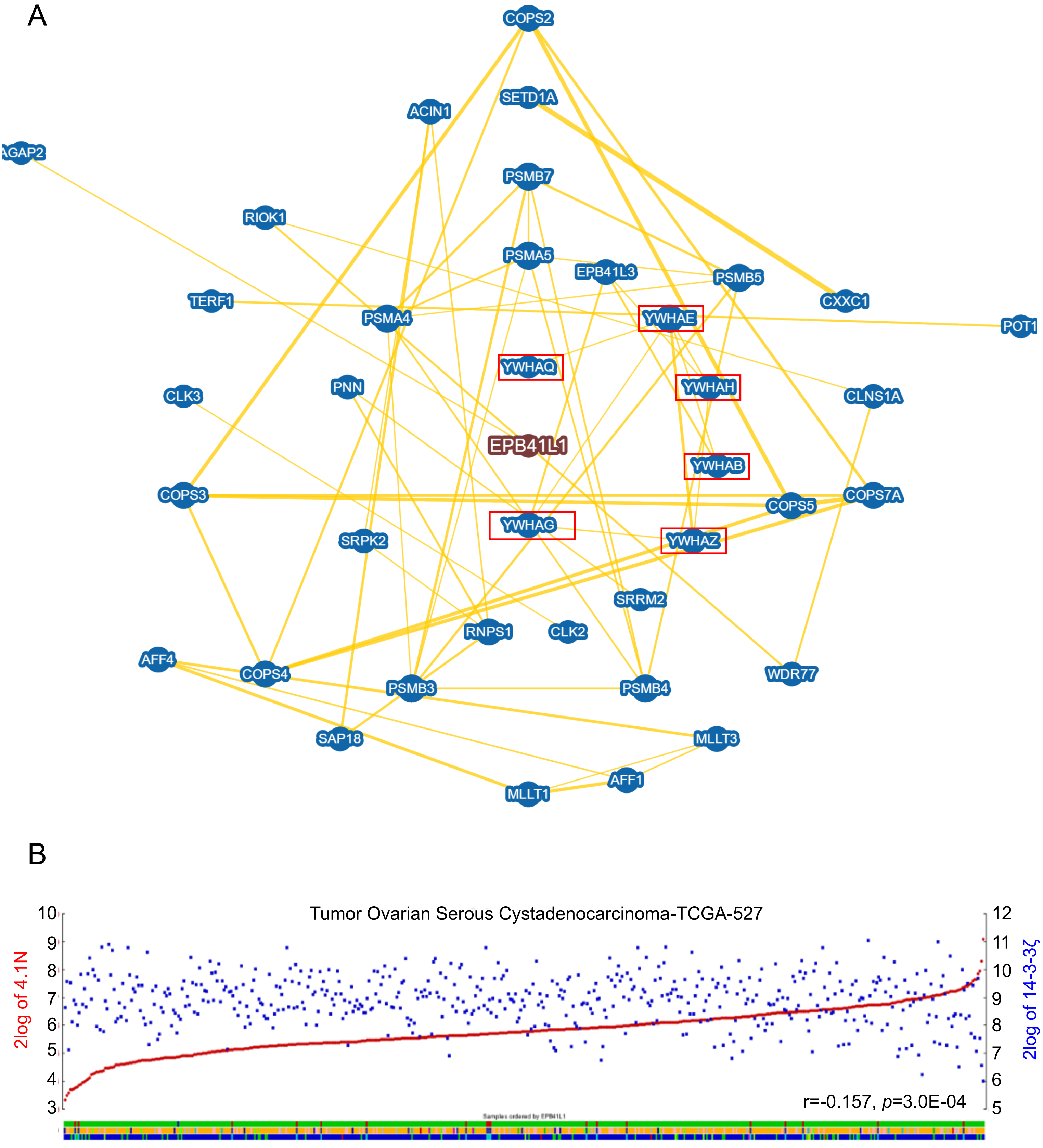
A
COPS2
SETD1A
ACIN1
AGAP2
PSMB7
RIOK1
PSMA5
EPB41L3
PSMB5
TERF1
PSMA4
YWHAE
CXXC1
POT1
YWHAQ
YWHAH
CLNS1A
CLK3
PNN
EPB41L1
YWHAB
COPS3
COPS5
COPS7A
YWHAG
SRPK2
YWHAZ
SRRM2
RNPS1
CLK2
AFF4
COPS4
PSMB3
PSMB4
WDR77
SAP18
MLLT3
AFF1
MLLT1
B
Tumor Ovarian Serous Cystadenocarcinoma-TCGA-527
2log of 4.1N
2log of 14-3-3
r=-0.157, p=3.0E-04
